# Supplementary material for: Evaluating the Performance of Integrated Management of Acute Malnutrition Programs in Somalia: A Systematic Review and Meta-Analysis
Source: Int J Environ Res Public Health. 2025 Mar 5;22(3):378. doi: 10.3390/ijerph22030378 (PMC11942193; doi:10.3390/ijerph22030378)

Appendix A

OTP Readmission

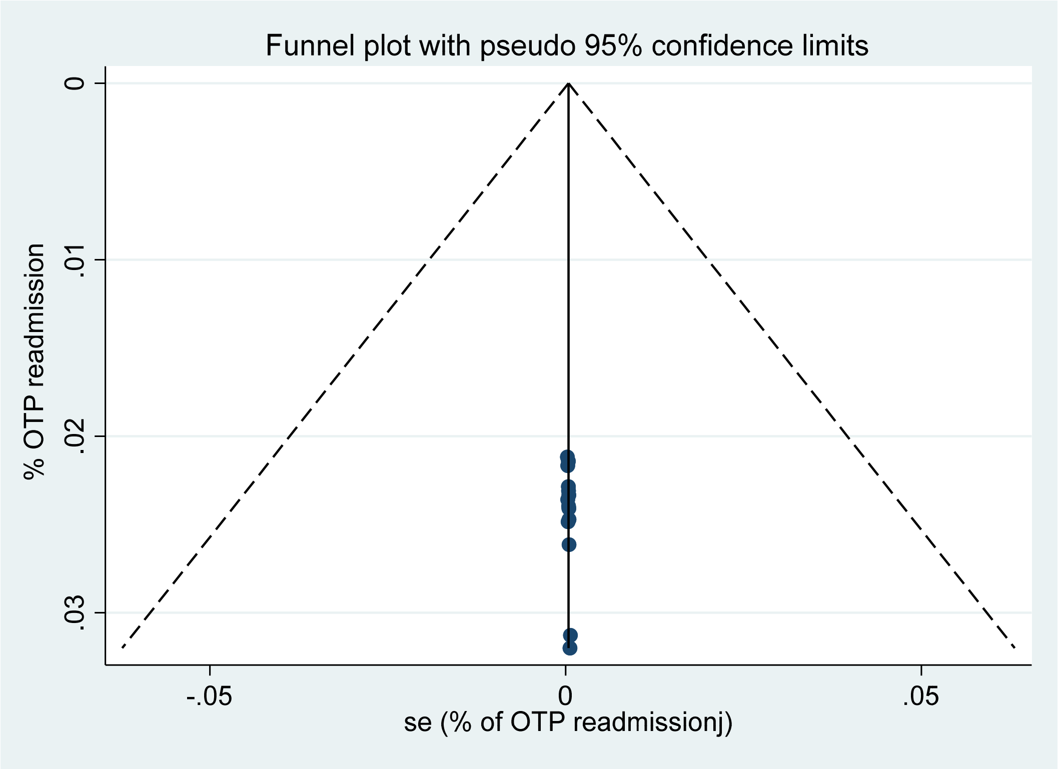

Egger's test

Std_Eff	Coefficient	Std. err.	t	P>t	95% CI	
Slope	-<br>0.0003545	0.0001451	-2.44	0.031	-0.00067	-0.0000384
Bias	0.031862	0.0060252	5.29	0.000	0.018734	0.0449897

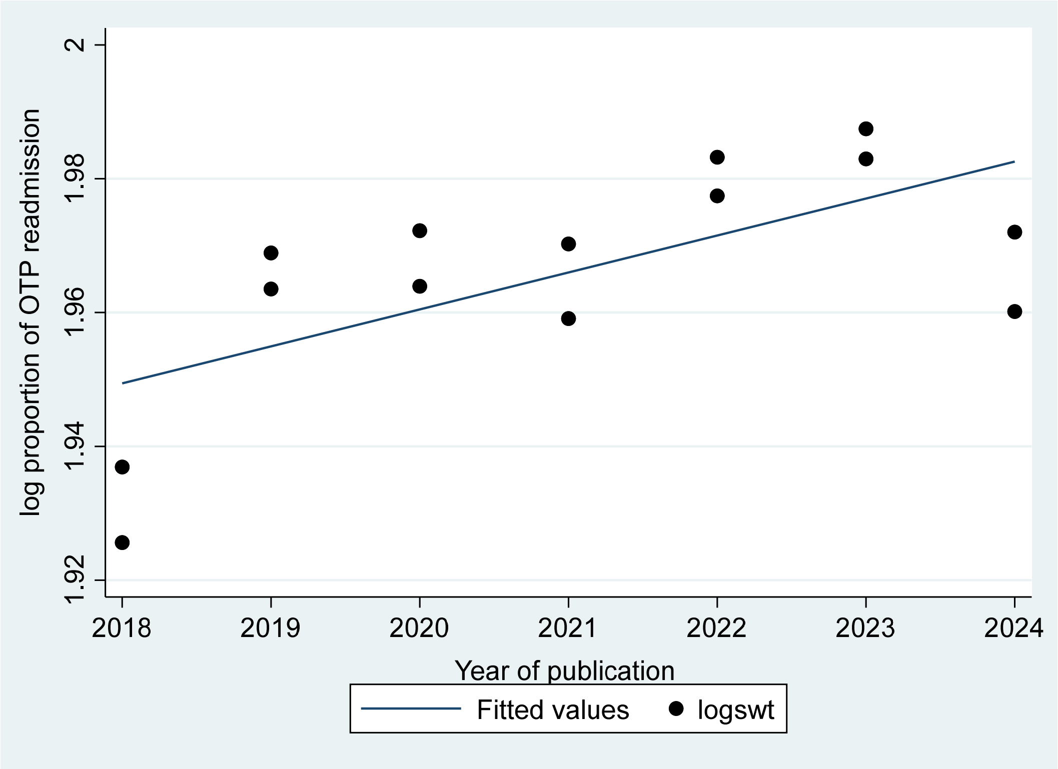

SC Readmission

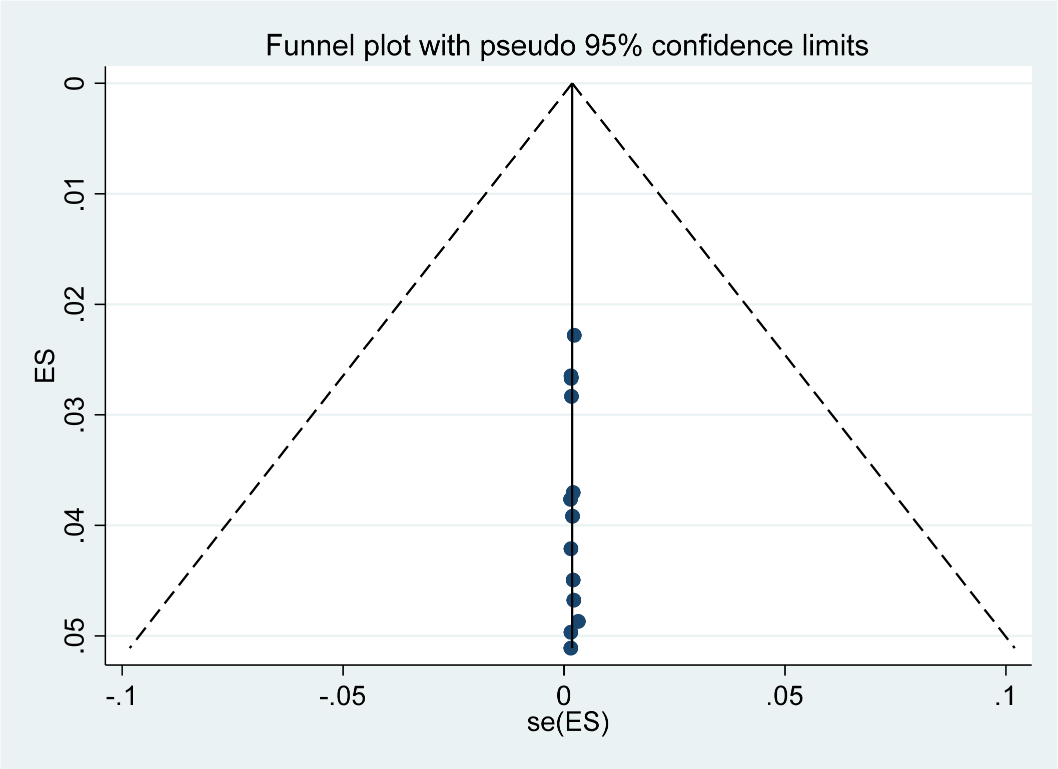

Egger's test

Std_Eff	Coefficient	Std. err.	t	P>t	95% CI	
Slope	0.0016677	0.0004177	3.99	0.002	0.000758	0.0025779
Bias	0.0054175	0.0123436	0.44	0.669	-0.02148	0.0323119

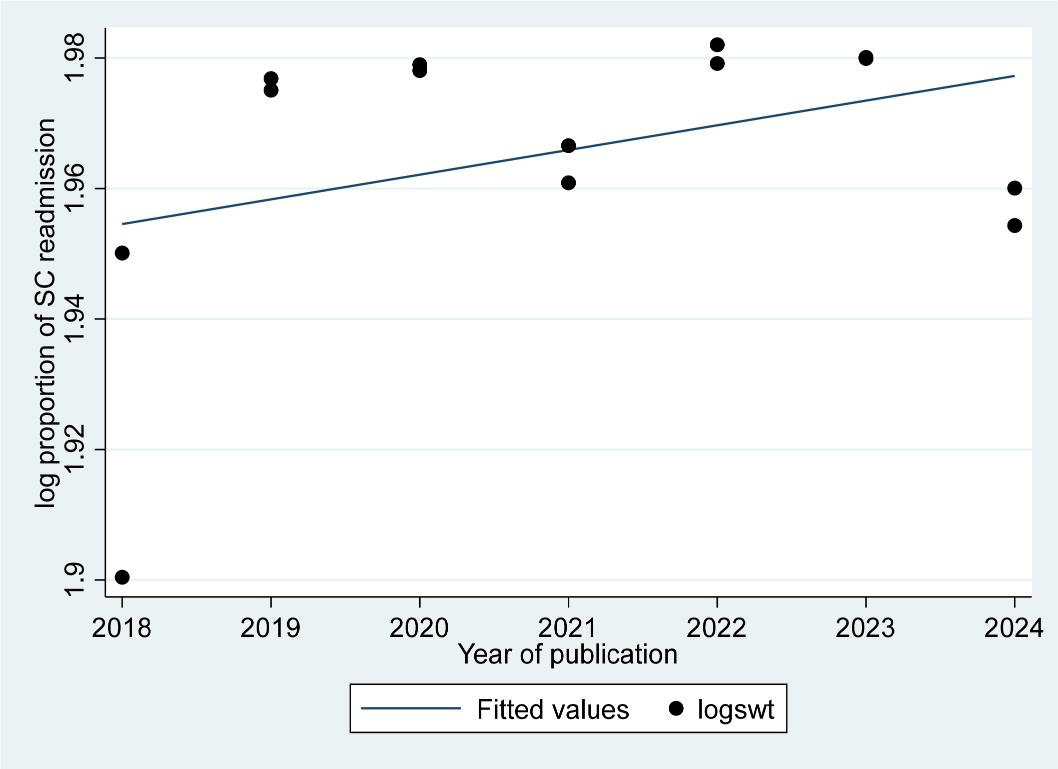

OTP recovery

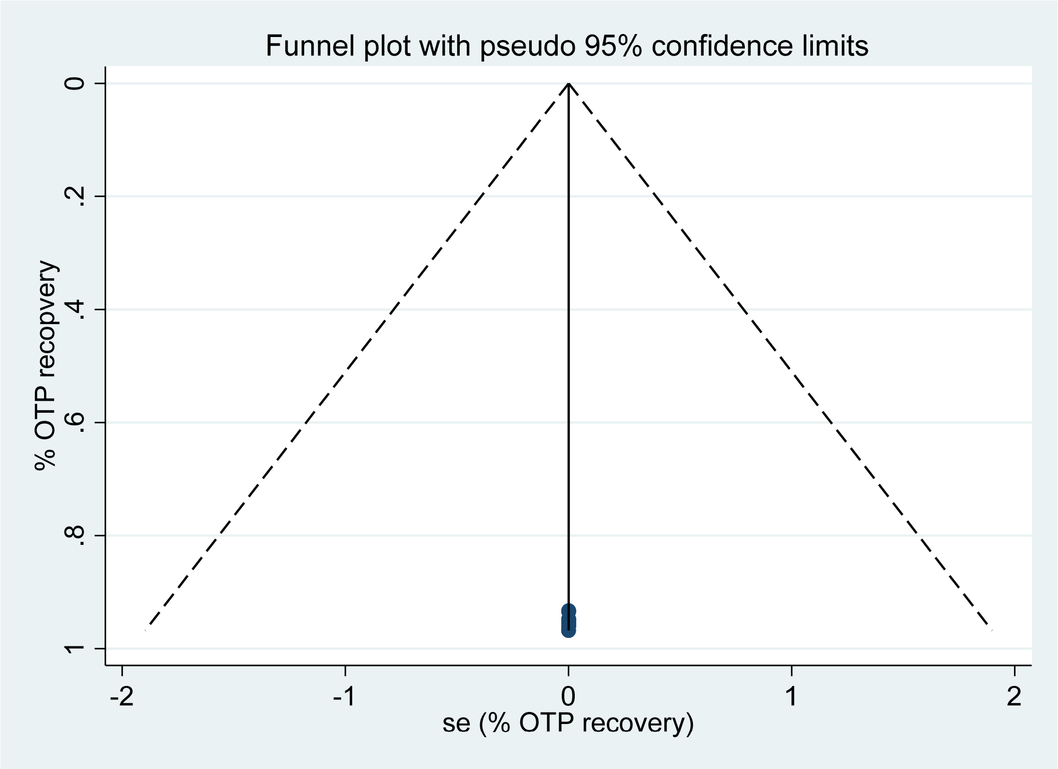

Egger's test

Std_Eff	Coefficient	Std. err.	t	P>t	95% CI	
Slope	0.0126816	0.0027798	4.56	0.001	0.006625	0.0187383
Bias	-0.0126317	0.0029148	-4.33	0.001	-0.01898	-0.0062808

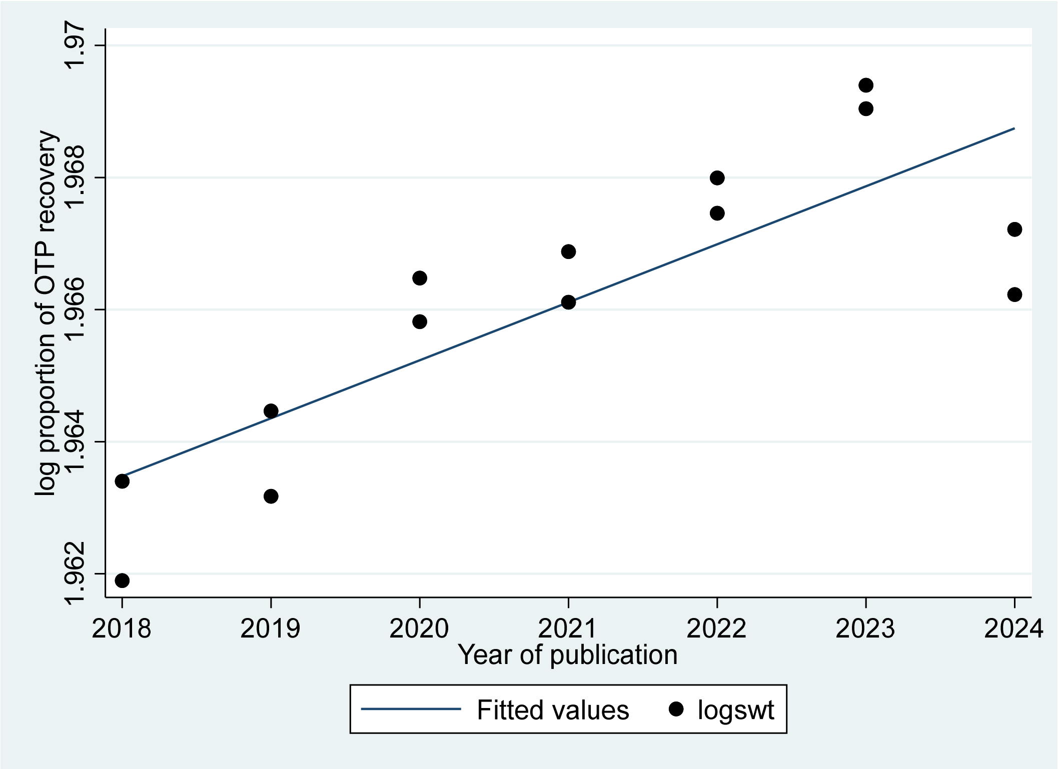

SC recovery

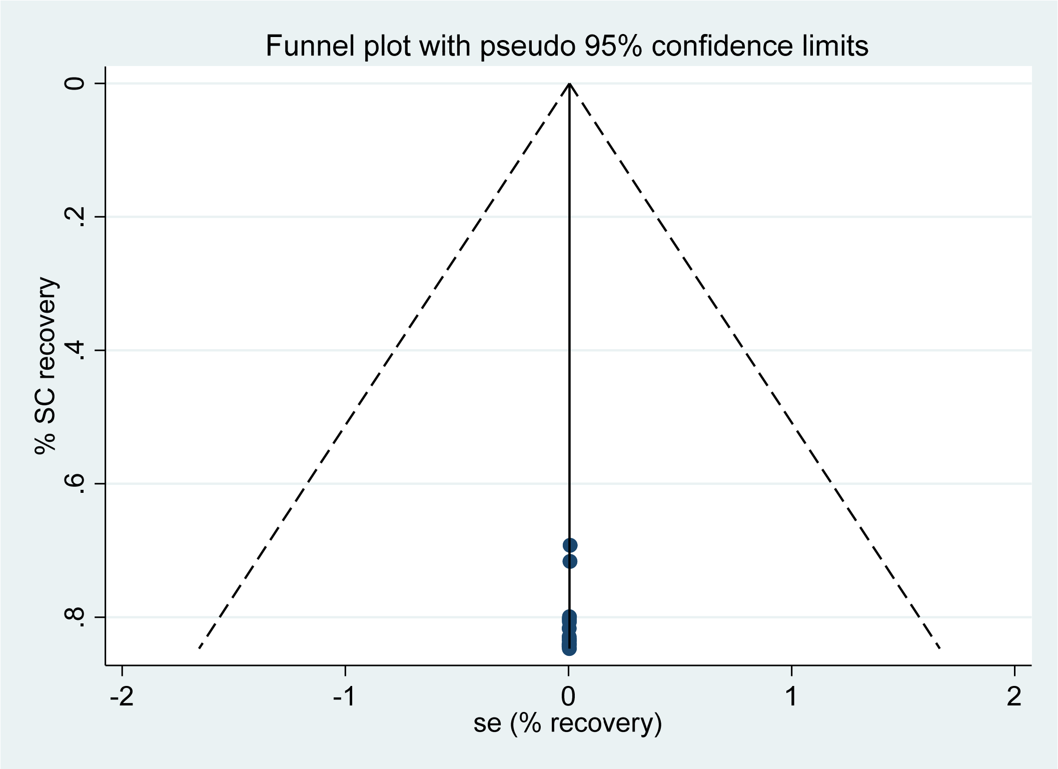

Egger's test

Std_Eff	Coefficient	Std. err.	t	P>t	95% CI	
Slope	0.0261113	0.0021267	12.28	0.000	0.021478	0.0307451
Bias	-0.0273386	0.0026479	-10.32	0.000	-0.03311	-0.0215692

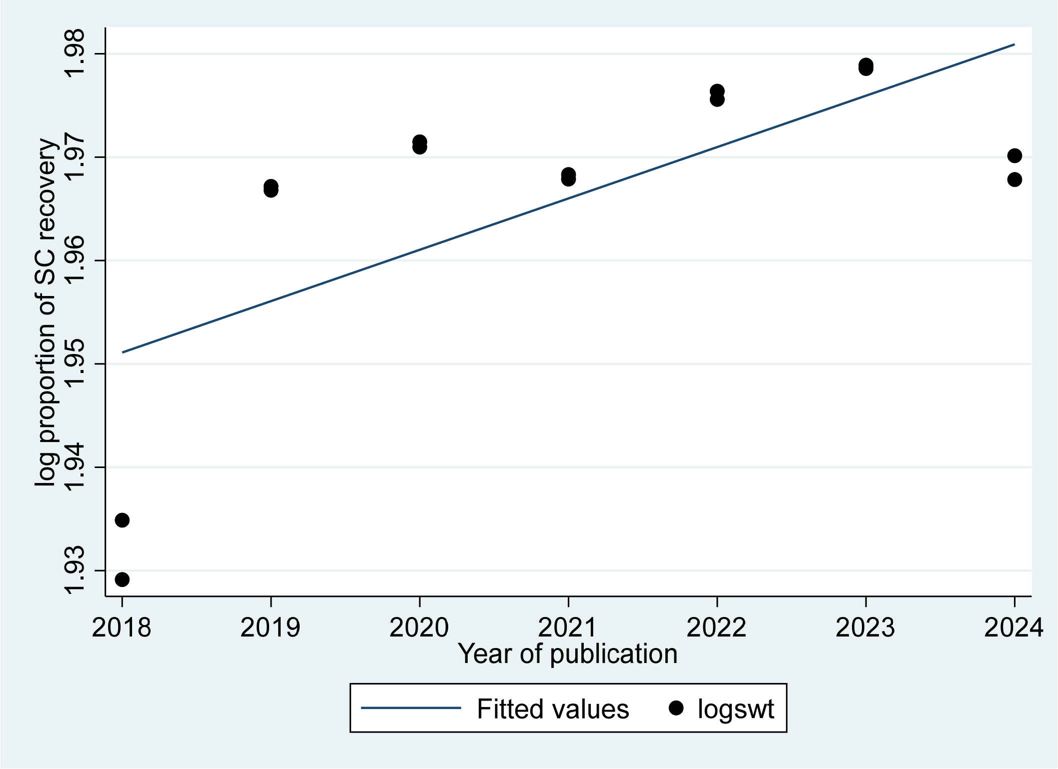

OTP deaths

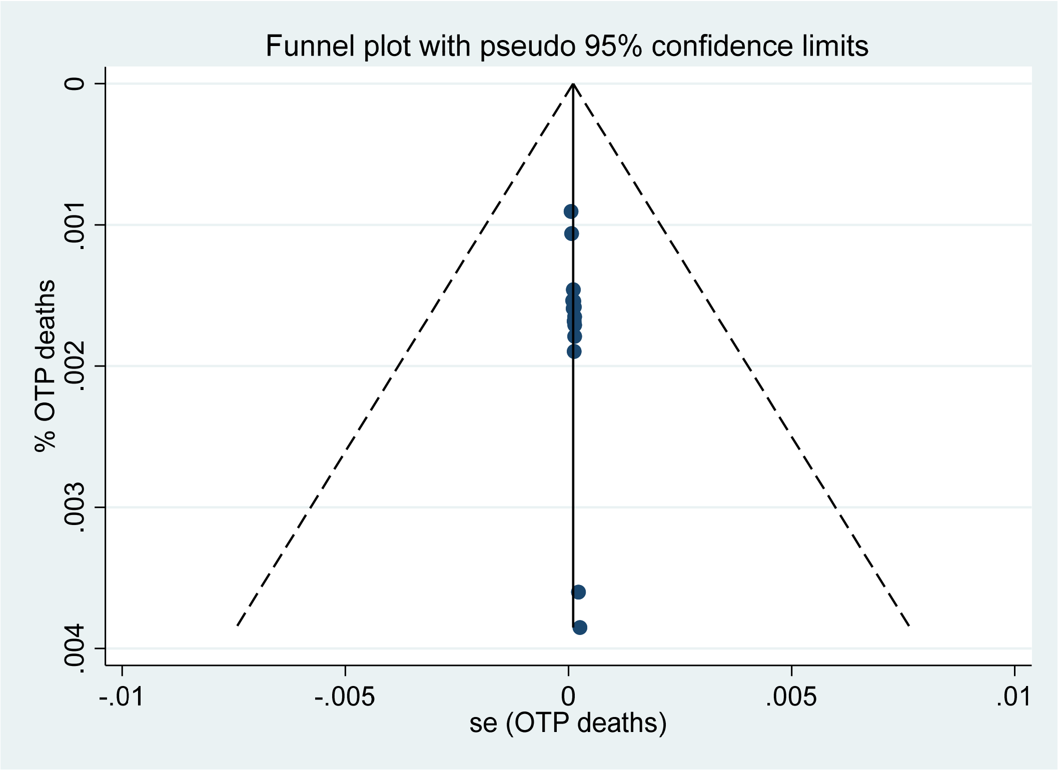

Egger's test

Std_Eff	Coefficient	Std. err.	t	P>t	95% CI	
Slope	-3.01E-06	0.0000106	-0.28	0.782	-2.6E-05	0.0000201
Bias	0.0732704	0.0069658	10.52	0.000	0.058093	0.0884475

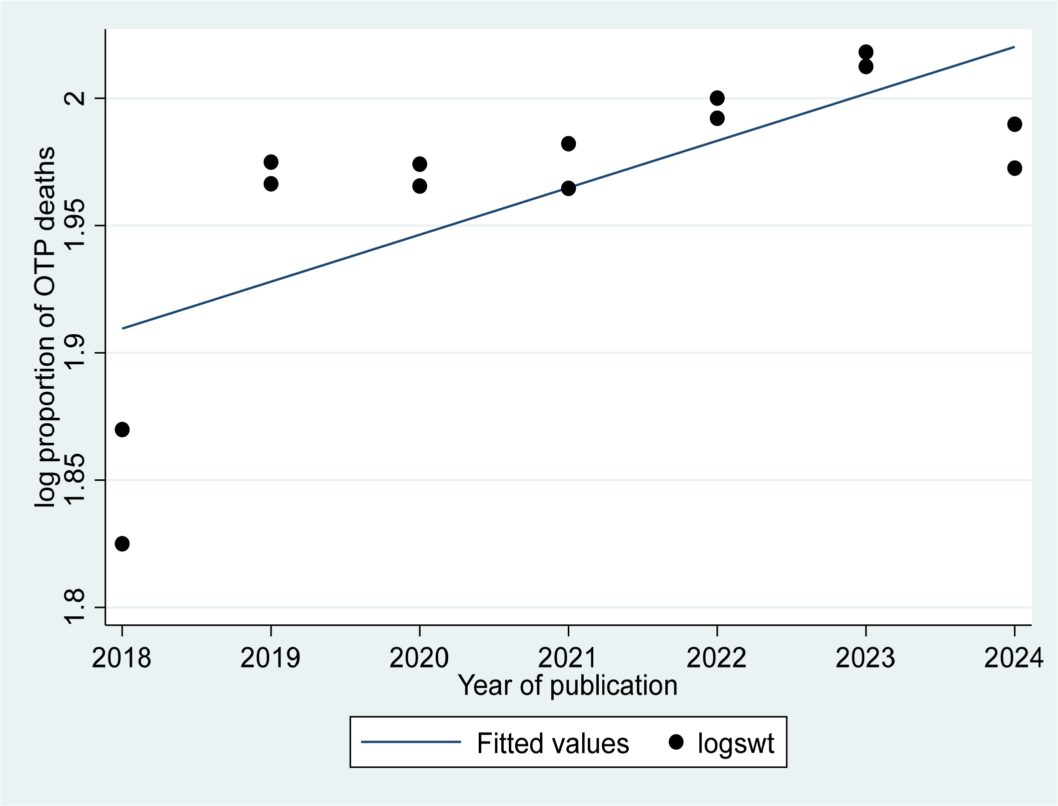

SC deaths

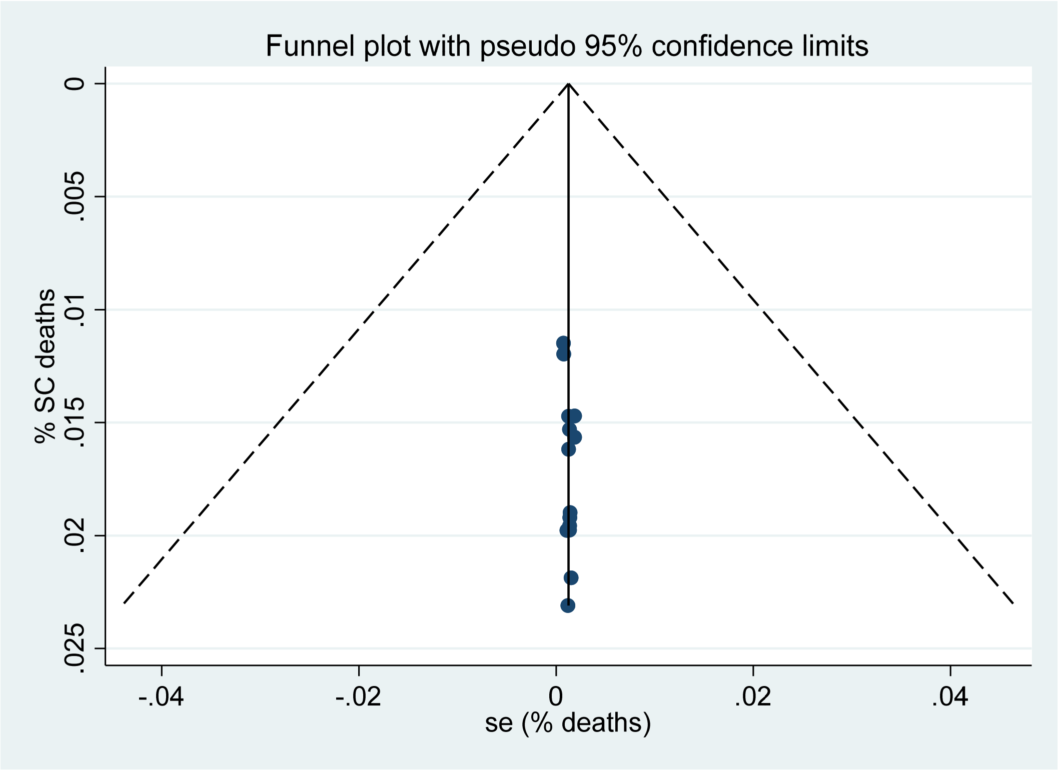

Egger's test

Std_Eff	Coefficient	Std. err.	t	P>t	95% CI	
Slope	0.0004404	0.0004519	0.97	0.349	-0.00054	0.001425
Bias	0.0513255	0.0278506	1.84	0.090	-0.00936	0.1120067

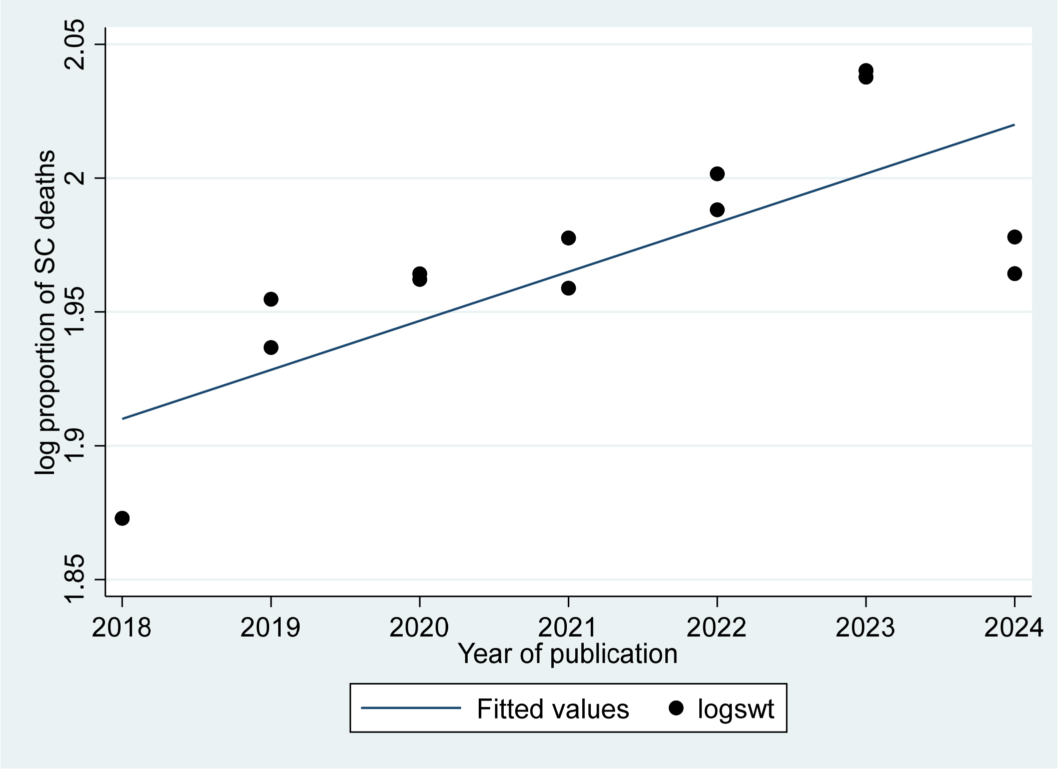

OTP Defaulters

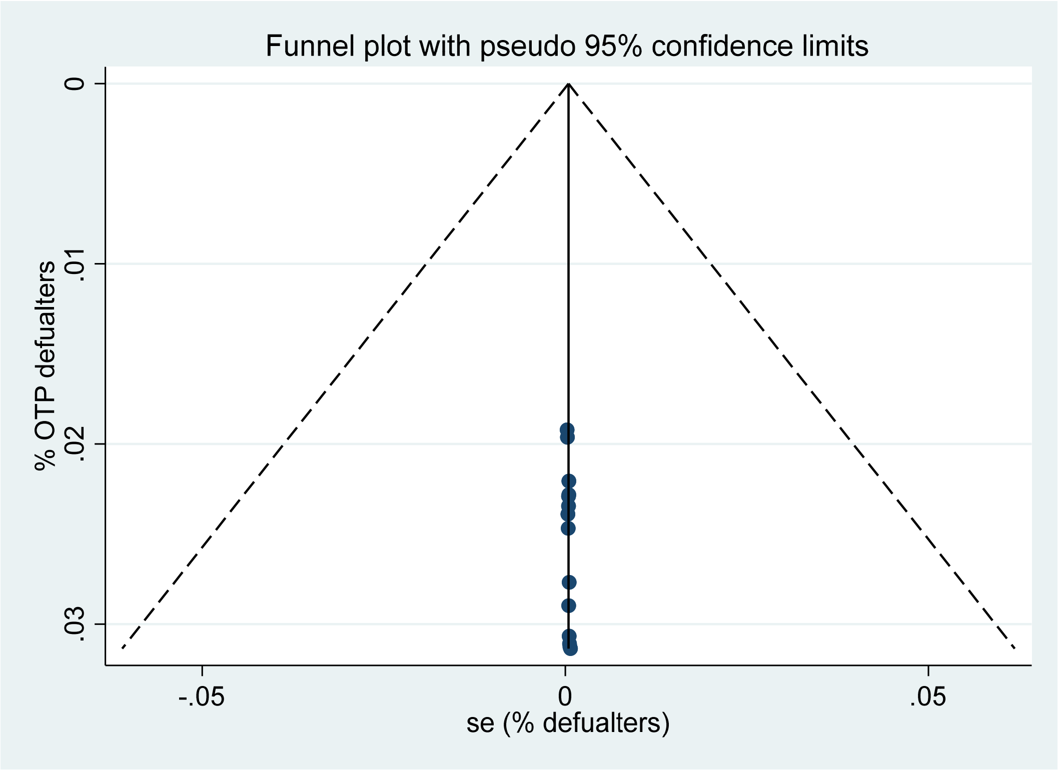

Egger's test

Std_Eff	Coefficient	Std. err.	t	P>t	95% CI	
Slope	-0.0001753	0.0001102	-1.59	0.138	-0.00042	0.0000649
Bias	0.0254606	0.0044718	5.69	0.000	0.015717	0.0352037

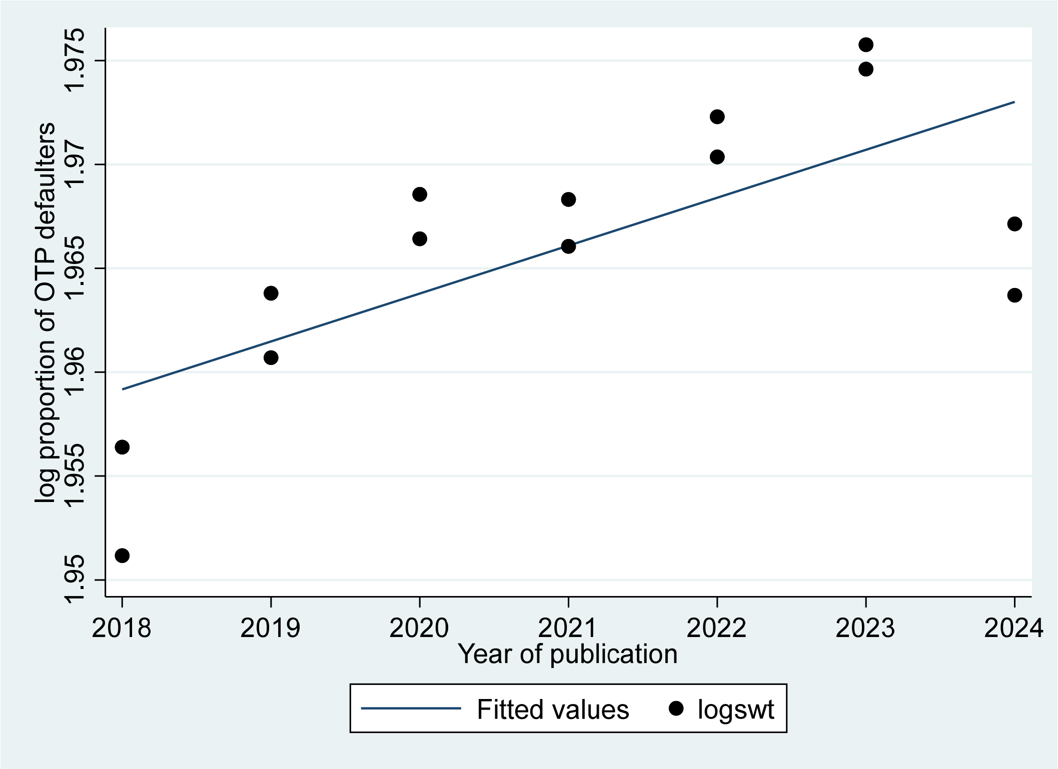

SC defaulters

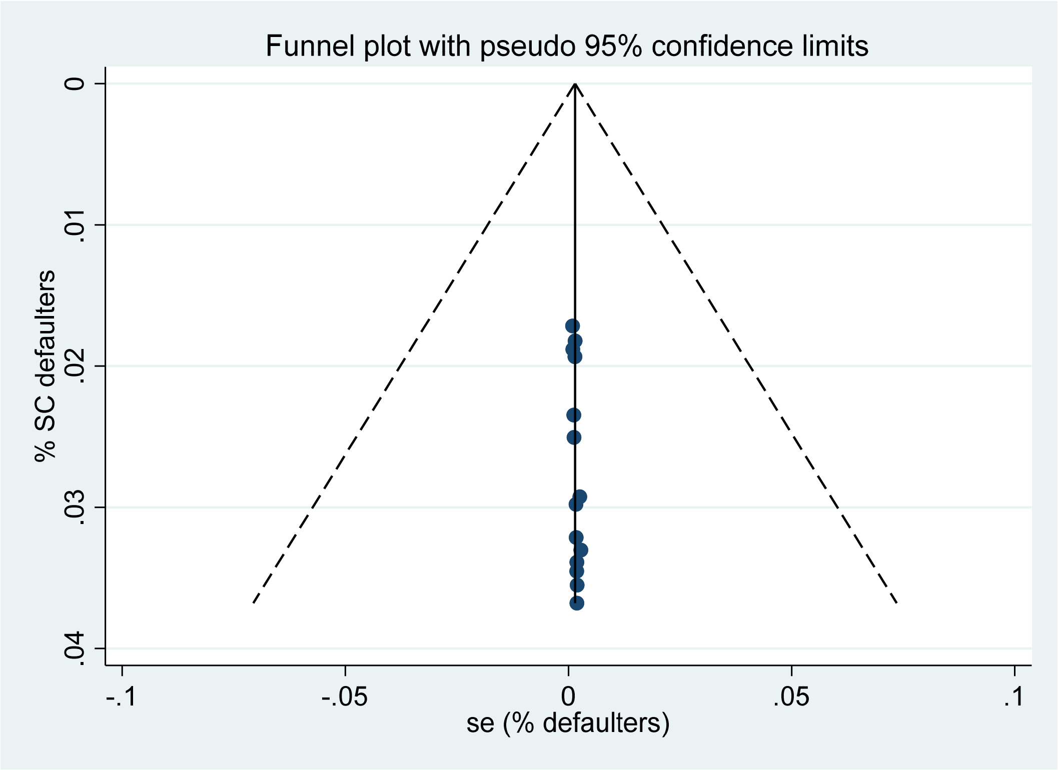

Egger's test

Std_Eff	Coefficient	Std. err.	t	P>t	95% CI	
Slope	0.0001427	0.0003525	0.4	0.693	-0.00063	0.0009107
Bias	0.0555273	0.0142085	3.91	0.002	0.02457	0.086485

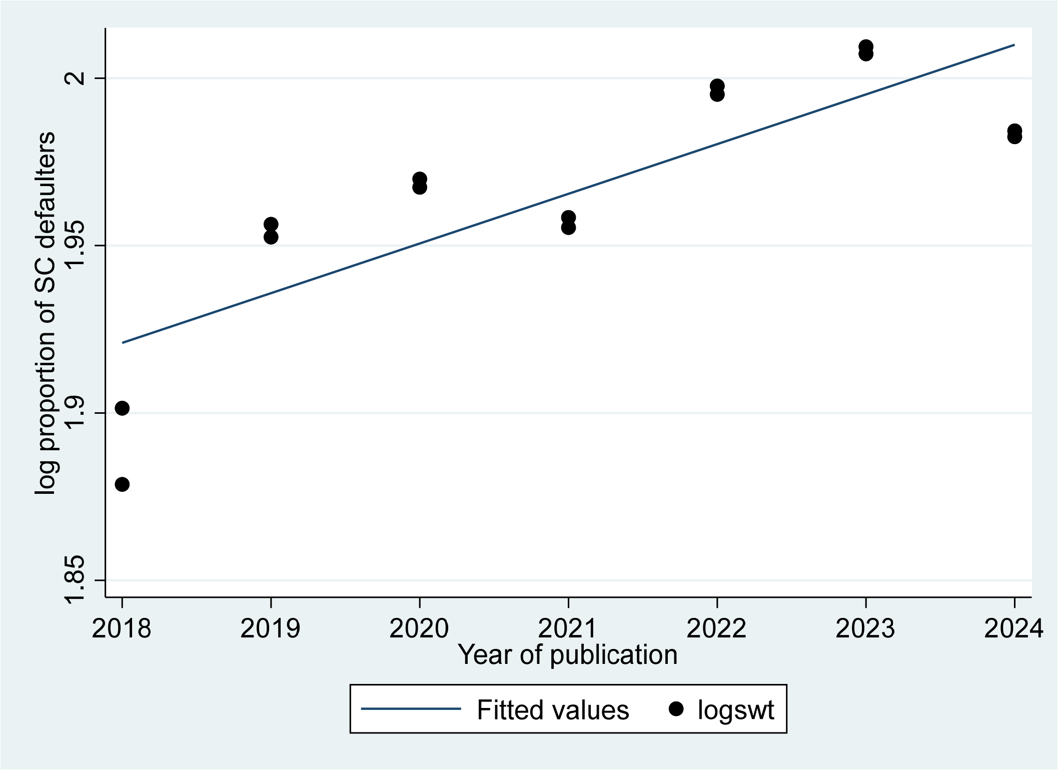

OTP non-recovery

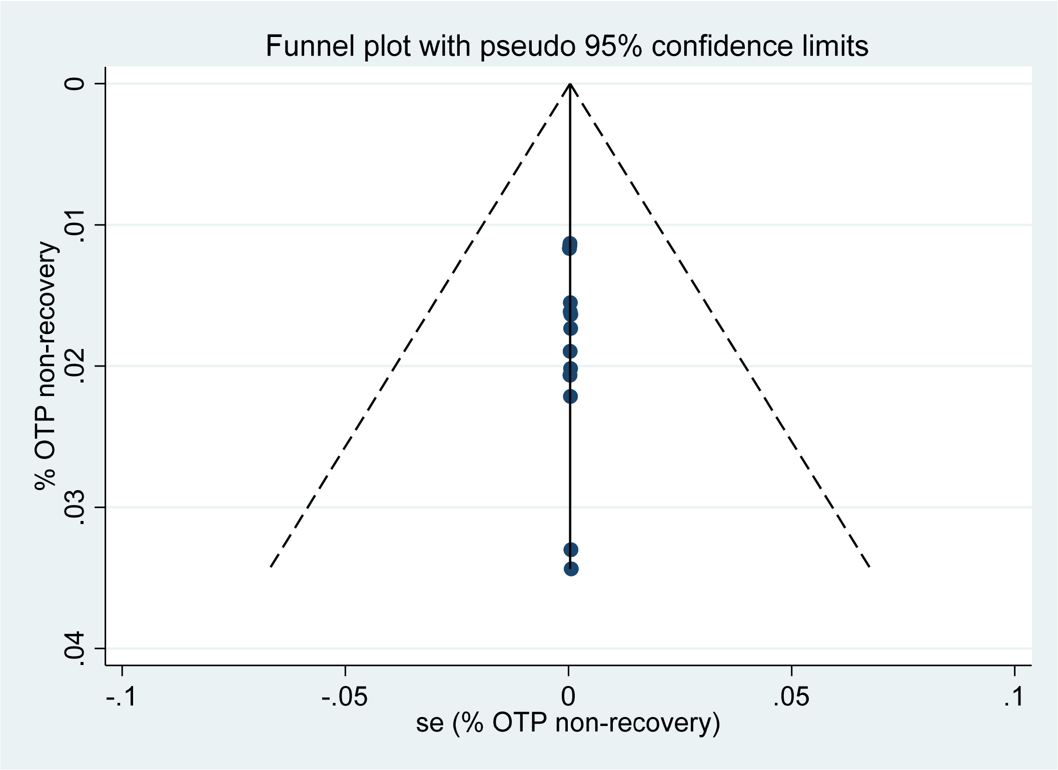

Egger's test

Std_Eff	Coefficient	Std. err.	t	P>t	95% CI	
Slope	0.0001132	0.0000695	1.63	0.129	-3.8E-05	0.0002646
Bias	0.015741	0.0044179	3.56	0.004	0.006115	0.0253668

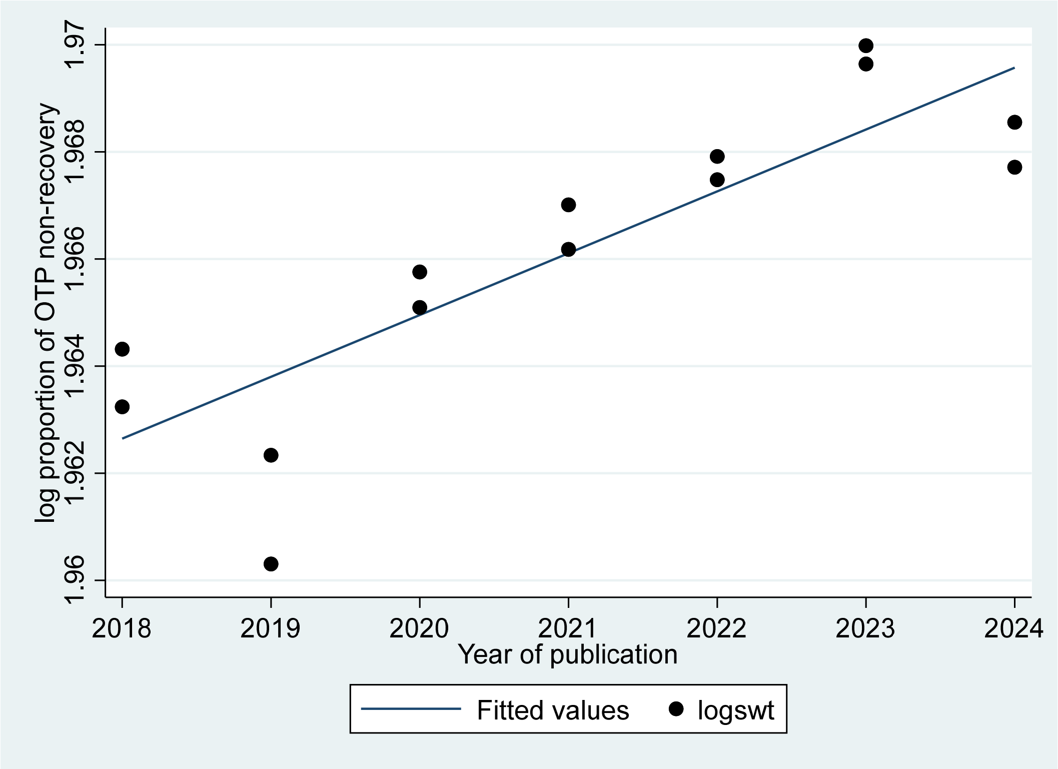

SC non-recovery

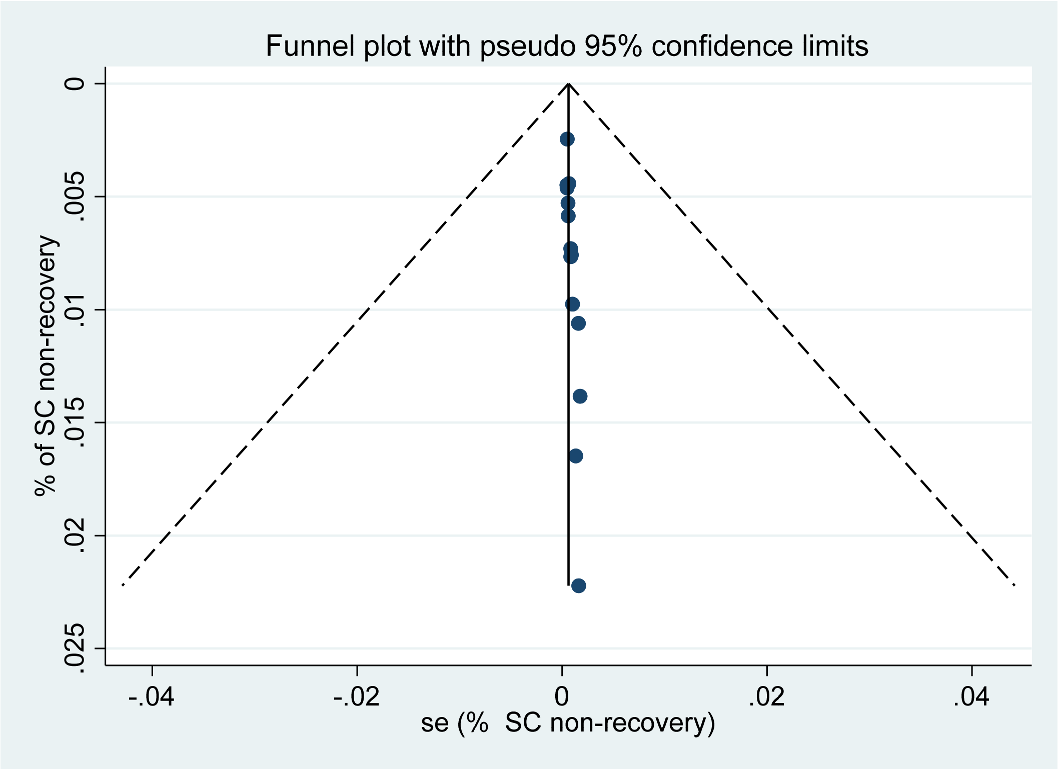

Egger's test

Std_Eff	Coefficient	Std. err.	t	P>t	95% CI	
Slope	0.0002478	0.0000705	3.52	0.004	9.42E-05	0.0004014
Bias	0.0791205	0.0128149	6.17	0.000	0.051199	0.1070418

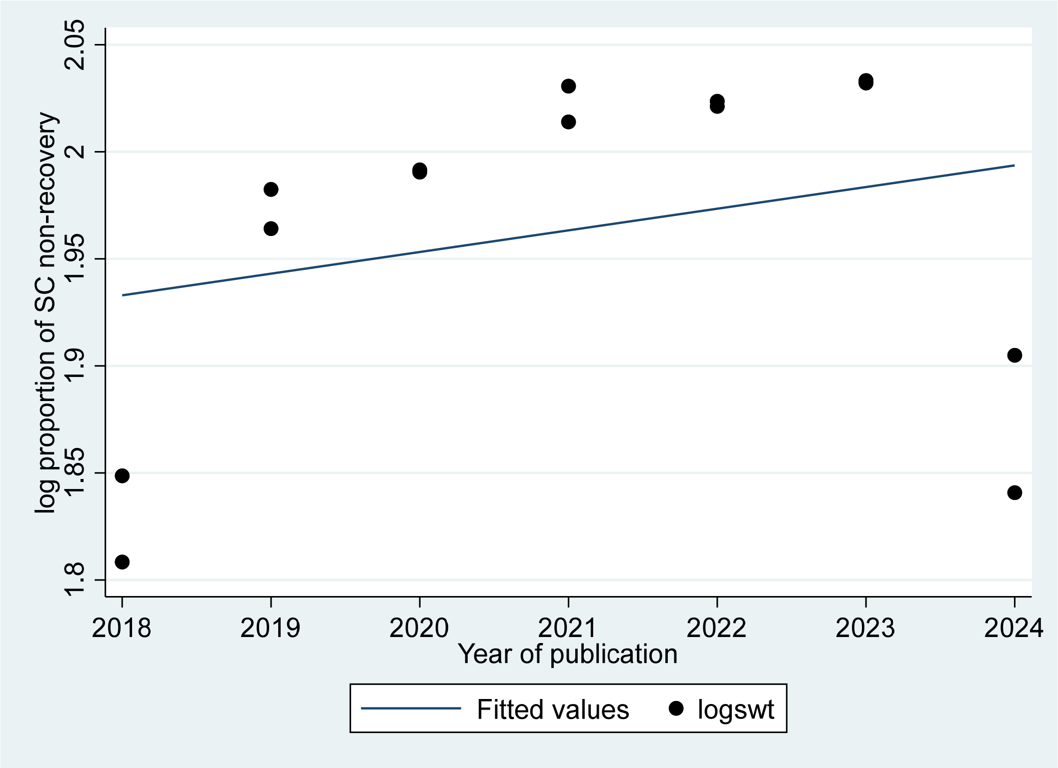

SC transfers

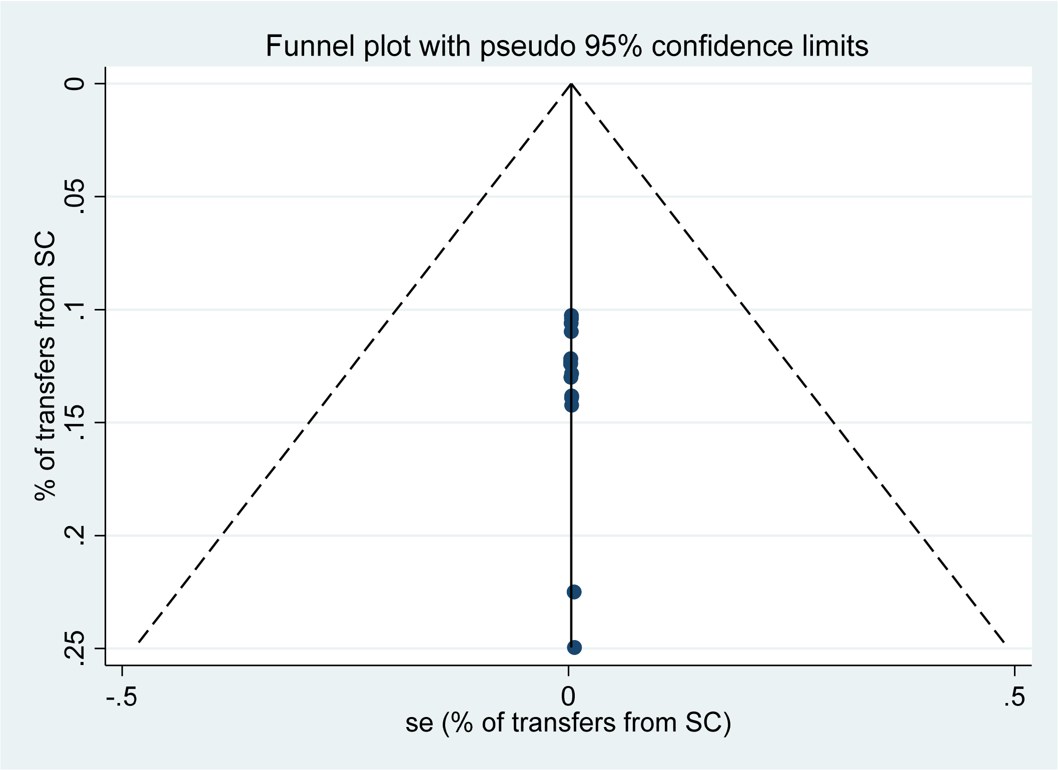

Egger's test

Std_Eff	Coefficient	Std. err.	t	P>t	95% CI	
Slope	0.000334	0.0007077	0.47	0.645	-0.00121	0.001876
Bias	0.0229001	0.0055708	4.11	0.001	0.010762	0.0350378

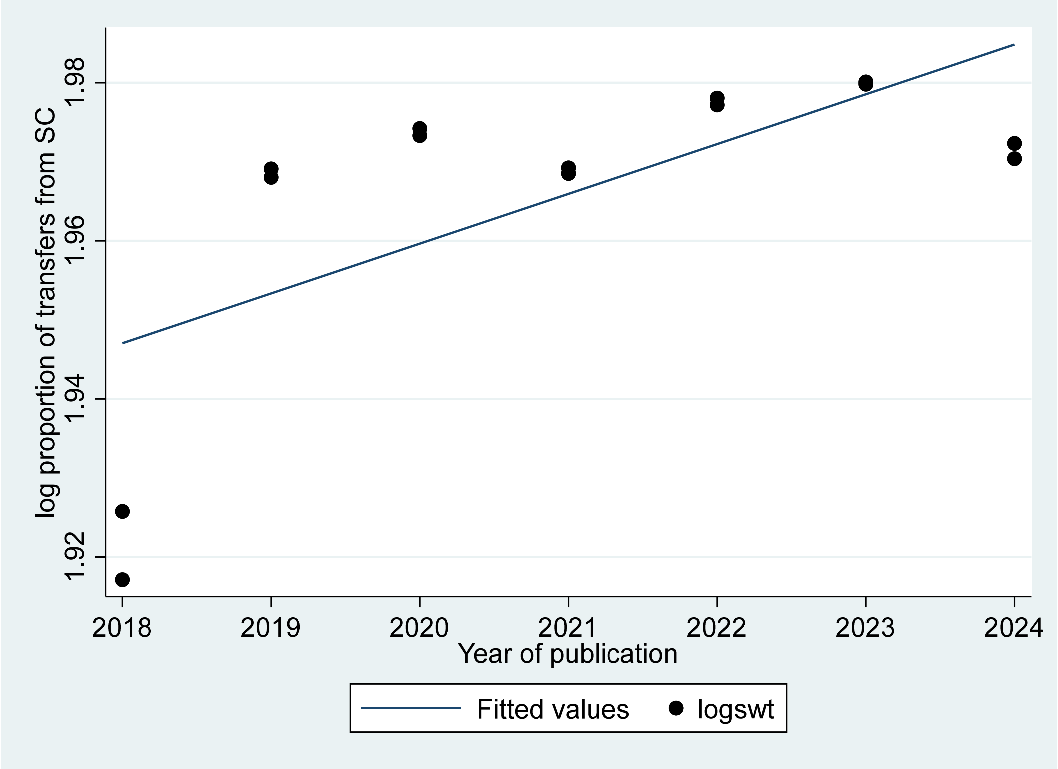

Supplement: Supplementary file 1 [file ijerph-22-00378-s001.zip › Figure S1A Bias analysis 1.pdf]
