# Supplementary material for: Evaluating the Performance of Integrated Management of Acute Malnutrition Programs in Somalia: A Systematic Review and Meta-Analysis
Source: Int J Environ Res Public Health. 2025 Mar 5;22(3):378. doi: 10.3390/ijerph22030378 (PMC11942193; doi:10.3390/ijerph22030378)
Supplement: Supplementary file 1 [file ijerph-22-00378-s001.zip › Figure S1B Bias analysis 2.pdf]

```
-----------------------------------------------------------------------------
--------------

. metaprop otp_readmission otp_admissionall, by(sex) random
cimethod(exact) power(2) l
> abel(namevar=author, yearvar=year) xlab(0, 2, 3,  4,5) sortby(year
author) texts(100)
>   rflevel(70)

           Study     |      ES      [95% Conf. Interval]  % Weight
---------------------+-------------------------------------------------------
     Boys
UNICEF (2018)        |         3.13      3.00         3.26         6.86
UNICEF (2019)        |         2.41      2.32         2.50         7.12
UNICEF (2020)        |         2.33      2.24         2.42         7.13
UNICEF (2021)        |         2.61      2.52         2.71         7.09
UNICEF (2022)        |         2.48      2.42         2.55         7.22
UNICEF (2023)        |         2.36      2.30         2.42         7.26
UNICEF (2024)        |         2.47      2.38         2.57         7.10
 Sub-total           |
  Random pooled  ES  |         2.54      2.39         2.68        49.79
---------------------+-------------------------------------------------------
     Girls
UNICEF (2018)        |         3.20      3.08         3.32         6.94
UNICEF (2019)        |         2.40      2.31         2.48         7.16
UNICEF (2020)        |         2.14      2.06         2.22         7.19
UNICEF (2021)        |         2.31      2.23         2.39         7.17
UNICEF (2022)        |         2.17      2.11         2.23         7.27
UNICEF (2023)        |         2.12      2.07         2.17         7.30
UNICEF (2024)        |         2.29      2.21         2.36         7.19
 Sub-total           |
  Random pooled  ES  |         2.37      2.18         2.56        50.21
---------------------+-------------------------------------------------------
Overall              |
  Random pooled  ES  |         2.45      2.32         2.58       100.00
---------------------+-------------------------------------------------------

Test(s) of heterogeneity:
               Heterogeneity  degrees of
                 statistic     freedom           P       I^2**
Boys                  128.56      6            0.00     95.33%
Girls                 295.40      6            0.00     97.97%
Overall               532.25     13            0.00     97.56%
** I^2: the variation in ES attributable to heterogeneity)


Random: Test for heterogeneity between sub-groups:
                        1.84      1            0.17

Significance test(s) of  ES=0

Boys                  z= 33.57      p = 0.00
Girls                 z= 24.25      p = 0.00
Overall               z= 36.59      p = 0.00
-----------------------------------------------------------------------------

.
. ge logswt = log(_WT)

. ge logsees = log(_seES)

. **correct plot
```

```
. metabias6 _seES  _ES, graph(begg)

Note: default data input format (theta, se_theta) assumed.

Tests for Publication Bias

Begg's Test

  adj. Kendall's Score (P-Q) =        63
          Std. Dev. of Score =     18.27
           Number of Studies =        14
                         z  =      3.45
                   Pr > |z| =     0.001
                         z  =      3.39  (continuity corrected)
                   Pr > |z| =     0.001  (continuity corrected)

Egger's test
------------------------------------------------------------------------
-----
     Std_Eff | Coefficient  Std. err.      t    P>|t|     [95% conf.
interval]
-------------+----------------------------------------------------------
-----
       slope |  -.0003545   .0001451    -2.44   0.031    -.0006706
-.0000384
        bias |    .031862   .0060252     5.29   0.000     .0187344
.0449897
------------------------------------------------------------------------
-----

. metafunnel _seES  _ES, by(sex)

Note: default data input format (theta, se_theta) assumed.

. metabias _seES  _ES, egger

Note: data input format theta se_theta assumed

Egger's test for small-study effects:
Regress standard normal deviate of intervention
  effect estimate against its standard error

.
Number of studies =  14                              Root MSE      =
.0025
------------------------------------------------------------------------
-----
     Std_Eff | Coefficient  Std. err.      t    P>|t|     [95% conf.
interval]
-------------+----------------------------------------------------------
-----
       slope |  -.0003545   .0001451    -2.44   0.031    -.0006706
-.0000384
        bias |    .031862   .0060252     5.29   0.000     .0187344
.0449897
------------------------------------------------------------------------
-----

Test of H0: no small-study effects          P = 0.000

.
.
. graph twoway (lfit logswt year) (scatter logswt year), ///
```

```
>         xlabel(2024(1)2018) ///
>   ytitle(log proportion of XXX) ///
>   xtitle(Year of publication)

. pwcorr logswt year, sig

             |   logswt     year
-------------+------------------
      logswt |   1.0000 
             |
             |
        year |   0.6667   1.0000 
             |   0.0092
             |


.
. ///sc readmission////
>
.

. metaprop sc_readmission sc_admissionall, by(sex) random
cimethod(exact) power(2) lab
> el(namevar=author, yearvar=year)  xlab (0, 2, 3,  4,5) sortby(year
author) texts(100)
>   rflevel(70)

           Study     |       ES       [95% Conf. Interval]  % Weight
---------------------+---------------------------------------------------
     Boys
UNICEF (2018)        |          2.28      1.85         2.78         7.03
UNICEF (2019)        |          2.66      2.35         3.00         7.22
UNICEF (2020)        |          2.67      2.37         3.00         7.23
UNICEF (2021)        |          3.92      3.55         4.31         7.15
UNICEF (2022)        |          3.76      3.48         4.06         7.26
UNICEF (2023)        |          4.97      4.67         5.28         7.24
UNICEF (2024)        |          3.70      3.31         4.13         7.10
 Sub-total           |
  Random pooled  ES  |          3.43      2.72         4.14        50.22
---------------------+---------------------------------------------------
     Girls
UNICEF (2018)        |          4.87      4.26         5.54         6.69
UNICEF (2019)        |          2.83      2.51         3.18         7.21
UNICEF (2020)        |          2.65      2.35         2.97         7.24
UNICEF (2021)        |          4.49      4.10         4.91         7.11
UNICEF (2022)        |          4.21      3.91         4.53         7.24
UNICEF (2023)        |          5.11      4.81         5.42         7.24
UNICEF (2024)        |          4.68      4.25         5.13         7.06
 Sub-total           |
  Random pooled  ES  |          4.11      3.33         4.90        49.78
---------------------+---------------------------------------------------
Overall              |
  Random pooled  ES  |          3.77      3.25         4.29       100.00
---------------------+---------------------------------------------------

Test(s) of heterogeneity:
               Heterogeneity  degrees of
                 statistic     freedom           P       I^2**
Boys                   182.32      6            0.00     96.71%
Girls                  199.93      6            0.00     97.00%
Overall                405.55     13            0.00     96.79%
** I^2: the variation in ES attributable to heterogeneity)
```

```
Random: Test for heterogeneity between sub-groups:
                         1.60      1            0.21

Significance test(s) of  ES=0

Boys                     z= 9.47       p = 0.00
Girls                    z= 10.25      p = 0.00
Overall                  z= 14.20      p = 0.00
-------------------------------------------------------------------------

.
. ge logswt = log(_WT)

. ge logsees = log(_seES)

. **correct plot
. metabias6 _seES  _ES, graph(begg)

Note: default data input format (theta, se_theta) assumed.

Tests for Publication Bias

Begg's Test

  adj. Kendall's Score (P-Q) =       23
          Std. Dev. of Score =    18.27
           Number of Studies =       14
                          z  =     1.26
                 Pr > |z| =    0.208
                          z  =     1.20 (continuity corrected)
                 Pr > |z| =    0.228 (continuity corrected)

Egger's test
-------------------------------------------------------------------------
-----
     Std_Eff | Coefficient  Std. err.      t    P>|t|     [95% conf.
interval]
-------------+-----------------------------------------------------------
-----
       slope |   .0016677   .0004177     3.99   0.002     .0007576
.0025779
        bias |   .0054175   .0123436     0.44   0.669    -.0214769
.0323119
-------------------------------------------------------------------------
-----

. metafunnel _seES  _ES

Note: default data input format (theta, se_theta) assumed.

. metabias _seES  _ES, egger

Note: data input format theta se_theta assumed

Egger's test for small-study effects:
Regress standard normal deviate of intervention
  effect estimate against its standard error

.
Number of studies =  14                               Root MSE      =
.0123
-------------------------------------------------------------------------
-----
```

```
     Std_Eff | Coefficient  Std. err.      t    P>|t|     [95% conf.
interval]
-------------+------------------------------------------------------------
-----
       slope |   .0016677   .0004177     3.99   0.002     .0007576
.0025779
        bias |   .0054175   .0123436     0.44   0.669    -.0214769
.0323119
--------------------------------------------------------------------------
-----

Test of H0: no small-study effects          P = 0.669

.
.
. graph twoway (lfit logswt year) (scatter logswt year), ///
>         xlabel(2024(1)2018) ///
>   ytitle(log proportion of XXX) ///
>   xtitle(Year of publication)

. pwcorr logswt year, sig

             |   logswt     year
-------------+------------------
      logswt |   1.0000
             |
             |
        year |   0.3630   1.0000
             |   0.2021
             |

.
. ////OTP recovery/////
>
.
.

. metaprop otprecovery tot_otpexit, by(sex) random  cimethod(exact)
power(2) label(name
> var=author, yearvar=year)  xlab(0, 50, 60, 70, 80, 90) sortby(year
author) texts(100)
>   rflevel(70)

           Study     |      ES       [95% Conf. Interval]  % Weight
---------------------+----------------------------------------------------
     Boys
UNICEF (2018)        |        94.84     94.66        95.02        7.11
UNICEF (2019)        |        93.29     93.12        93.45        7.12
UNICEF (2020)        |        95.52     95.39        95.65        7.14
UNICEF (2021)        |        96.07     95.94        96.20        7.14
UNICEF (2022)        |        95.48     95.37        95.58        7.15
UNICEF (2023)        |        96.77     96.69        96.84        7.16
UNICEF (2024)        |        95.94     95.81        96.07        7.14
 Sub-total           |
  Random pooled  ES  |        95.42     94.65        96.19       49.98
---------------------+----------------------------------------------------
     Girls
UNICEF (2018)        |        94.78     94.61        94.94        7.12
UNICEF (2019)        |        93.46     93.31        93.61        7.13
UNICEF (2020)        |        95.25     95.13        95.37        7.15
UNICEF (2021)        |        95.94     95.82        96.06        7.15
UNICEF (2022)        |        95.39     95.29        95.49        7.16
UNICEF (2023)        |        96.82     96.76        96.89        7.17
```

```
UNICEF (2024)        |        95.81     95.70        95.92        7.15
 Sub-total           |
  Random pooled  ES  |        95.35     94.57        96.13       50.02
---------------------+------------------------------------------------------
Overall              |
  Random pooled  ES  |        95.39     94.87        95.90      100.00
---------------------+------------------------------------------------------

Test(s) of heterogeneity:
               Heterogeneity  degrees of
                 statistic     freedom          P       I^2**
Boys                 1711.54      6            0.00     99.65%
Girls                2181.68      6            0.00     99.72%
Overall              3893.54     13            0.00     99.67%
** I^2: the variation in ES attributable to heterogeneity)


Random: Test for heterogeneity between sub-groups:
                        0.01      1            0.91

Significance test(s) of  ES=0

Boys                 z= 243.20     p = 0.00
Girls                z= 239.73     p = 0.00
Overall              z= 362.29     p = 0.00
----------------------------------------------------------------------------

.
. ge logswt = log(_WT)

. ge logsees = log(_seES)

. **correct plot
. metabias6 _seES  _ES, graph(begg)

Note: default data input format (theta, se_theta) assumed.

Tests for Publication Bias

Begg's Test

  adj. Kendall's Score (P-Q) =     -43
          Std. Dev. of Score =   18.27
           Number of Studies =     14
                          z  =   -2.35
                    Pr > |z| =   0.019
                          z  =    2.30 (continuity corrected)
                    Pr > |z| =   0.021 (continuity corrected)

Egger's test
----------------------------------------------------------------------------
-----
     Std_Eff | Coefficient  Std. err.      t    P>|t|     [95% conf.
interval]
-------------+--------------------------------------------------------------
-----
       slope |   .0126816   .0027798     4.56   0.001     .0066249
.0187383
        bias |  -.0126317   .0029148    -4.33   0.001    -.0189825
-.0062808
----------------------------------------------------------------------------
-----
```

```
. metafunnel _seES  _ES

Note: default data input format (theta, se_theta) assumed.

. metabias _seES  _ES, egger

Note: data input format theta se_theta assumed

Egger's test for small-study effects:
Regress standard normal deviate of intervention
  effect estimate against its standard error

.
Number of studies =  14                              Root MSE      =
1.2e-04
------------------------------------------------------------------------
-----
     Std_Eff | Coefficient  Std. err.      t    P>|t|     [95% conf.
interval]
-------------+----------------------------------------------------------
-----
       slope |   .0126816   .0027798     4.56   0.001     .0066249
.0187383
        bias |  -.0126317   .0029148    -4.33   0.001    -.0189825
-.0062808
------------------------------------------------------------------------
-----

Test of H0: no small-study effects          P = 0.001

.
.
. graph twoway (lfit logswt year) (scatter logswt year), ///
>        xlabel(2024(1)2018) ///
>   ytitle(log proportion of XXX) ///
>   xtitle(Year of publication)

. pwcorr logswt year, sig

             |   logswt     year
-------------+------------------
      logswt |   1.0000 
             |
             |
        year |   0.8267   1.0000 
             |   0.0003
             |

.
.
.
. ///SC rfecovery///
>

. metaprop sc_recovery tot_sc_exit , by(sex) random  cimethod(exact)
power(2) label(nam
> evar=author, yearvar=year)  xlab(0, 50, 60, 70, 80, 90) sortby(year
author) texts(100
> )  rflevel(70)

           Study     |      ES       [95% Conf. Interval]  % Weight
---------------------+----------------------------------------------------
     Boys
```

```
UNICEF (2018)        |        69.21     67.78        70.62         6.88
UNICEF (2019)        |        79.92     79.09        80.73         7.15
UNICEF (2020)        |        83.36     82.63        84.08         7.18
UNICEF (2021)        |        80.36     79.57        81.14         7.16
UNICEF (2022)        |        82.92     82.33        83.50         7.22
UNICEF (2023)        |        84.06     83.54        84.57         7.23
UNICEF (2024)        |        84.03     83.22        84.82         7.16
 Sub-total           |
  Random pooled  ES  |        80.61     78.20        83.03        49.97
---------------------+---------------------------------------------------
     Girls
UNICEF (2018)        |        71.63     70.28        72.96         6.92
UNICEF (2019)        |        80.68     79.86        81.48         7.15
UNICEF (2020)        |        83.45     82.73        84.15         7.18
UNICEF (2021)        |        80.15     79.35        80.94         7.16
UNICEF (2022)        |        81.66     81.05        82.26         7.21
UNICEF (2023)        |        84.36     83.85        84.86         7.23
UNICEF (2024)        |        84.69     83.93        85.42         7.17
 Sub-total           |
  Random pooled  ES  |        81.00     78.76        83.23        50.03
---------------------+---------------------------------------------------
Overall              |
  Random pooled  ES  |        80.81     79.25        82.37       100.00
---------------------+---------------------------------------------------

Test(s) of heterogeneity:
               Heterogeneity  degrees of
                 statistic     freedom           P       I^2**
Boys                   466.78      6           0.00     98.71%
Girls                  410.28      6           0.00     98.54%
Overall                877.24     13           0.00     98.52%
** I^2: the variation in ES attributable to heterogeneity)


Random: Test for heterogeneity between sub-groups:
                         0.05      1           0.82

Significance test(s) of  ES=0

Boys                   z= 65.46      p = 0.00
Girls                  z= 70.96      p = 0.00
Overall                z= 101.32     p = 0.00
-------------------------------------------------------------------------


.
. ge logswt = log(_WT)

. ge logsees = log(_seES)

. **correct plot
. metabias6 _seES  _ES, graph(begg)

Note: default data input format (theta, se_theta) assumed.

Tests for Publication Bias

Begg's Test

  adj. Kendall's Score (P-Q) =     -55
          Std. Dev. of Score =   18.27
           Number of Studies =      14
                          z  =   -3.01
                   Pr > |z| =   0.003
```

```
                    z  =    2.96  (continuity corrected)
              Pr > |z| =   0.003  (continuity corrected)

Egger's test
------------------------------------------------------------------------
-----
     Std_Eff | Coefficient  Std. err.      t    P>|t|     [95% conf.
interval]
-------------+----------------------------------------------------------
-----
       slope |   .0261113   .0021267    12.28   0.000     .0214776
.0307451
        bias |  -.0273386   .0026479   -10.32   0.000    -.0331079
-.0215692
------------------------------------------------------------------------
-----

. metafunnel _seES  _ES

Note: default data input format (theta, se_theta) assumed.

. metabias _seES  _ES, egger

Note: data input format theta se_theta assumed

Egger's test for small-study effects:
Regress standard normal deviate of intervention
  effect estimate against its standard error

.
Number of studies =  14                                Root MSE      =
6.1e-04
------------------------------------------------------------------------
-----
     Std_Eff | Coefficient  Std. err.      t    P>|t|     [95% conf.
interval]
-------------+----------------------------------------------------------
-----
       slope |   .0261113   .0021267    12.28   0.000     .0214776
.0307451
        bias |  -.0273386   .0026479   -10.32   0.000    -.0331079
-.0215692
------------------------------------------------------------------------
-----

Test of H0: no small-study effects          P = 0.000

.
.
. graph twoway (lfit logswt year) (scatter logswt year), ///
>        xlabel(2024(1)2018) ///
>   ytitle(log proportion of XXX) ///
>   xtitle(Year of publication)

. pwcorr logswt year, sig

             |   logswt     year
-------------+------------------
      logswt |   1.0000
             |
             |
        year |   0.6863   1.0000
             |   0.0067
```

```
            |

.
.
. ////OTP deaths
>

. metaprop otp_died tot_otpexit , by(sex) random  cimethod(exact)
power(2) label(nameva
> r=author, yearvar=year)  xlab(0, 0.1, 0.2, 0.3, 0.4) sortby(year
author) texts(100)
> rflevel(70)

           Study     |      ES       [95% Conf. Interval]  % Weight
---------------------+---------------------------------------------------
     Boys
UNICEF (2018)        |        0.39      0.34          0.44         6.20
UNICEF (2019)        |        0.17      0.14          0.19         7.14
UNICEF (2020)        |        0.18      0.15          0.21         7.14
UNICEF (2021)        |        0.17      0.14          0.20         7.13
UNICEF (2022)        |        0.16      0.14          0.18         7.33
UNICEF (2023)        |        0.11      0.09          0.12         7.48
UNICEF (2024)        |        0.16      0.13          0.19         7.19
 Sub-total           |
  Random pooled  ES  |        0.18      0.14          0.23        49.62
---------------------+---------------------------------------------------
     Girls
UNICEF (2018)        |        0.36      0.32          0.41         6.49
UNICEF (2019)        |        0.17      0.14          0.20         7.21
UNICEF (2020)        |        0.19      0.17          0.22         7.20
UNICEF (2021)        |        0.15      0.13          0.18         7.26
UNICEF (2022)        |        0.15      0.14          0.17         7.39
UNICEF (2023)        |        0.09      0.08          0.10         7.52
UNICEF (2024)        |        0.15      0.13          0.17         7.31
 Sub-total           |
  Random pooled  ES  |        0.18      0.13          0.22        50.38
---------------------+---------------------------------------------------
Overall              |
  Random pooled  ES  |        0.18      0.15          0.21       100.00
---------------------+---------------------------------------------------

Test(s) of heterogeneity:
               Heterogeneity  degrees of
                 statistic     freedom           P       I^2**
Boys                   132.84       6           0.00     95.48%
Girls                  195.98       6           0.00     96.94%
Overall                334.05      13           0.00     96.11%
** I^2: the variation in ES attributable to heterogeneity)


Random: Test for heterogeneity between sub-groups:
                        0.06       1            0.81

Significance test(s) of  ES=0

Boys                    z= 8.52       p = 0.00
Girls                   z= 7.80       p = 0.00
Overall                 z= 12.17      p = 0.00
-------------------------------------------------------------------------

.
. ge logswt = log(_WT)
```

```
. ge logsees = log(_seES)

. **correct plot
. metabias6 _seES  _ES, graph(begg)

Note: default data input format (theta, se_theta) assumed.

Tests for Publication Bias

Begg's Test

  adj. Kendall's Score (P-Q) =      65
          Std. Dev. of Score =   18.27
           Number of Studies =      14
                          z  =    3.56
                    Pr > |z| =   0.000
                          z  =    3.50 (continuity corrected)
                    Pr > |z| =   0.000 (continuity corrected)

Egger's test
------------------------------------------------------------------------------
     Std_Eff | Coefficient  Std. err.      t    P>|t|     [95% conf.
interval]
-------------+----------------------------------------------------------------
       slope |  -3.01e-06   .0000106    -0.28   0.782    -.0000262
.0000201
        bias |   .0732704   .0069658    10.52   0.000     .0580933
.0884475
------------------------------------------------------------------------------

. metafunnel _seES  _ES

Note: default data input format (theta, se_theta) assumed.

. metabias _seES  _ES, egger

Note: data input format theta se_theta assumed

Egger's test for small-study effects:
Regress standard normal deviate of intervention
  effect estimate against its standard error

.
Number of studies =  14                                Root MSE      =
.0083
------------------------------------------------------------------------------
     Std_Eff | Coefficient  Std. err.      t    P>|t|     [95% conf.
interval]
-------------+----------------------------------------------------------------
       slope |  -3.01e-06   .0000106    -0.28   0.782    -.0000262
.0000201
        bias |   .0732704   .0069658    10.52   0.000     .0580933
.0884475
------------------------------------------------------------------------------

Test of H0: no small-study effects          P = 0.000
```

```
.
.
. graph twoway (lfit logswt year) (scatter logswt year), ///
>         xlabel(2024(1)2018) ///
>   ytitle(log proportion of XXX) ///
>   xtitle(Year of publication)

. pwcorr logswt year, sig

             |   logswt     year
-------------+------------------
      logswt |   1.0000
             |
             |
        year |   0.7196   1.0000
             |   0.0037
             |

.
. /// SC deaths///
>

. metaprop sc_died tot_sc_exit , by(sex) random  cimethod(exact) power(2)
label(namevar
> =author, yearvar=year)  xlab(0, 1, 1.5, 2, 2.5) sortby(year author)
texts(100)  rflev
> el(70)

           Study     |      ES       [95% Conf. Interval]  % Weight
---------------------+---------------------------------------------------
     Boys
UNICEF (2018)        |         1.47      1.13          1.89         6.51
UNICEF (2019)        |         1.90      1.63          2.20         7.06
UNICEF (2020)        |         1.96      1.70          2.24         7.11
UNICEF (2021)        |         1.62      1.38          1.89         7.23
UNICEF (2022)        |         1.98      1.77          2.20         7.40
UNICEF (2023)        |         1.20      1.05          1.36         7.67
UNICEF (2024)        |         1.53      1.28          1.82         7.13
 Sub-total           |
  Random pooled  ES  |         1.66      1.40          1.93        50.11
---------------------+---------------------------------------------------
     Girls
UNICEF (2018)        |         1.56      1.22          1.98         6.51
UNICEF (2019)        |         2.19      1.90          2.51         6.94
UNICEF (2020)        |         1.98      1.72          2.26         7.13
UNICEF (2021)        |         1.92      1.66          2.21         7.09
UNICEF (2022)        |         2.31      2.08          2.56         7.30
UNICEF (2023)        |         1.15      1.01          1.30         7.69
UNICEF (2024)        |         1.47      1.23          1.74         7.23
 Sub-total           |
  Random pooled  ES  |         1.79      1.42          2.17        49.89
---------------------+---------------------------------------------------
Overall              |
  Random pooled  ES  |         1.73      1.51          1.95       100.00
---------------------+---------------------------------------------------

Test(s) of heterogeneity:
              Heterogeneity  degrees of
                statistic     freedom           P       I^2**
Boys                  50.95      6            0.00     88.22%
Girls                 99.80      6            0.00     93.99%
Overall              151.71     13            0.00     91.43%
** I^2: the variation in ES attributable to heterogeneity)
```

```
Random: Test for heterogeneity between sub-groups:
                         0.31      1            0.57

Significance test(s) of  ES=0

Boys                     z= 12.26      p = 0.00
Girls                    z= 9.32       p = 0.00
Overall                  z= 15.52      p = 0.00
-------------------------------------------------------------------------

.
. ge logswt = log(_WT)

. ge logsees = log(_seES)

. **correct plot
. metabias6 _seES  _ES, graph(begg)

Note: default data input format (theta, se_theta) assumed.

Tests for Publication Bias

Begg's Test

  adj. Kendall's Score (P-Q) =       5
          Std. Dev. of Score =   18.27
           Number of Studies =      14
                          z  =    0.27
                    Pr > |z| =   0.784
                          z  =    0.22 (continuity corrected)
                    Pr > |z| =   0.827 (continuity corrected)

Egger's test
-------------------------------------------------------------------------
-----
     Std_Eff | Coefficient  Std. err.      t    P>|t|     [95% conf.
interval]
-------------+-----------------------------------------------------------
-----
       slope |   .0004404   .0004519     0.97   0.349    -.0005443
.001425
        bias |   .0513255   .0278506     1.84   0.090    -.0093557
.1120067
-------------------------------------------------------------------------
-----

. metafunnel _seES  _ES

Note: default data input format (theta, se_theta) assumed.

. metabias _seES  _ES, egger

Note: data input format theta se_theta assumed

Egger's test for small-study effects:
Regress standard normal deviate of intervention
  effect estimate against its standard error

.
Number of studies =  14                                 Root MSE      =
.0217
```

```
------------------------------------------------------------------------
-----
     Std_Eff | Coefficient  Std. err.      t    P>|t|     [95% conf.
interval]
-------------+----------------------------------------------------------
-----
       slope |   .0004404   .0004519     0.97   0.349    -.0005443
.001425
        bias |   .0513255   .0278506     1.84   0.090    -.0093557
.1120067
------------------------------------------------------------------------
-----

Test of H0: no small-study effects          P = 0.090

.
.
. graph twoway (lfit logswt year) (scatter logswt year), ///
>        xlabel(2024(1)2018) ///
>   ytitle(log proportion of XXX) ///
>   xtitle(Year of publication)

. pwcorr logswt year, sig

             |   logswt     year
-------------+------------------
      logswt |   1.0000 
             |
             |
        year |   0.7778   1.0000 
             |   0.0011
             |

.
.
. ///OTP defaulters
>


. metaprop otp_defaulters tot_otpexit , by(sex) random  cimethod(exact)
power(2) label(
> namevar=author, yearvar=year)  xlab(0, 1, 2, 3,  4) sortby(year author)
texts(100)  r
> flevel(70)

           Study     |      ES       [95% Conf. Interval]  % Weight
---------------------+--------------------------------------------------
     Boys
UNICEF (2018)        |        3.14      3.00         3.28         7.04
UNICEF (2019)        |        3.11      3.00         3.23         7.10
UNICEF (2020)        |        2.28      2.19         2.38         7.15
UNICEF (2021)        |        2.21      2.11         2.30         7.14
UNICEF (2022)        |        2.47      2.39         2.55         7.17
UNICEF (2023)        |        1.96      1.90         2.02         7.20
UNICEF (2024)        |        2.77      2.66         2.87         7.13
 Sub-total           |
  Random pooled  ES  |        2.56      2.23         2.89        49.93
---------------------+--------------------------------------------------
     Girls
UNICEF (2018)        |        3.13      3.00         3.26         7.07
UNICEF (2019)        |        3.07      2.96         3.17         7.13
UNICEF (2020)        |        2.34      2.26         2.43         7.16
UNICEF (2021)        |        2.29      2.20         2.38         7.16
```

```
UNICEF (2022)        |         2.39      2.32         2.46        7.19
UNICEF (2023)        |         1.92      1.87         1.97        7.21
UNICEF (2024)        |         2.90      2.81         2.99        7.15
 Sub-total           |
  Random pooled  ES  |         2.57      2.23         2.92       50.07
---------------------+----------------------------------------------------
Overall              |
  Random pooled  ES  |         2.57      2.34         2.79      100.00
---------------------+----------------------------------------------------

Test(s) of heterogeneity:
               Heterogeneity  degrees of
                 statistic     freedom          P       I^2**
Boys                    535.72      6            0.00     98.88%
Girls                   734.48      6            0.00     99.18%
Overall                1270.40     13            0.00     98.98%
** I^2: the variation in ES attributable to heterogeneity)


Random: Test for heterogeneity between sub-groups:
                         0.00      1            0.95

Significance test(s) of  ES=0

Boys                     z= 15.31      p = 0.00
Girls                    z= 14.72      p = 0.00
Overall                  z= 22.40      p = 0.00
--------------------------------------------------------------------------

.
. ge logswt = log(_WT)

. ge logsees = log(_seES)

. **correct plot
. metabias6 _seES  _ES, graph(begg)

Note: default data input format (theta, se_theta) assumed.

Tests for Publication Bias

Begg's Test

  adj. Kendall's Score (P-Q) =      55
          Std. Dev. of Score =   18.27
           Number of Studies =      14
                          z  =    3.01
                Pr > |z| =   0.003
                          z  =    2.96 (continuity corrected)
                Pr > |z| =   0.003 (continuity corrected)

Egger's test
--------------------------------------------------------------------------
-----
     Std_Eff | Coefficient  Std. err.      t    P>|t|     [95% conf.
interval]
-------------+------------------------------------------------------------
-----
       slope |  -.0001753   .0001102    -1.59   0.138    -.0004155
.0000649
        bias |   .0254606   .0044718     5.69   0.000     .0157174
.0352037
--------------------------------------------------------------------------
```

```
-----

. metafunnel _seES  _ES

Note: default data input format (theta, se_theta) assumed.

. metabias _seES  _ES, egger

Note: data input format theta se_theta assumed

Egger's test for small-study effects:
Regress standard normal deviate of intervention
  effect estimate against its standard error

.
Number of studies =  14                                Root MSE      =
.0028
------------------------------------------------------------------------
-----
     Std_Eff | Coefficient  Std. err.      t    P>|t|     [95% conf.
interval]
-------------+----------------------------------------------------------
-----
       slope |  -.0001753   .0001102    -1.59   0.138    -.0004155
.0000649
        bias |   .0254606   .0044718     5.69   0.000     .0157174
.0352037
------------------------------------------------------------------------
-----

Test of H0: no small-study effects          P = 0.000

.
.
. graph twoway (lfit logswt year) (scatter logswt year), ///
>         xlabel(2024(1)2018) ///
>   ytitle(log proportion of XXX) ///
>   xtitle(Year of publication)

. pwcorr logswt year, sig

             |   logswt     year
-------------+------------------
      logswt |   1.0000
             |
             |
        year |   0.7100   1.0000
             |   0.0044
             |

.
. /// SC defaulter///


. metaprop sc_defaulters tot_sc_exit , by(sex) random  cimethod(exact)
power(2) label(n
> amevar=author, yearvar=year)  xlab(0, 1, 2, 3,  4) sortby(year author)
texts(100)  rf
> level(70)

           Study     |      ES       [95% Conf. Interval]  % Weight
---------------------+------------------------------------------------
     Boys
```

```
UNICEF (2018)        |       3.30      2.78          3.89          6.54
UNICEF (2019)        |       3.39      3.03          3.78          7.07
UNICEF (2020)        |       2.98      2.66          3.33          7.17
UNICEF (2021)        |       3.68      3.32          4.07          7.07
UNICEF (2022)        |       2.35      2.12          2.59          7.37
UNICEF (2023)        |       1.88      1.70          2.08          7.44
UNICEF (2024)        |       1.82      1.54          2.13          7.26
 Sub-total           |
  Random pooled  ES  |       2.75      2.21          3.30         49.93
---------------------+---------------------------------------------------
     Girls
UNICEF (2018)        |       2.93      2.45          3.47          6.70
UNICEF (2019)        |       3.55      3.18          3.95          7.05
UNICEF (2020)        |       3.21      2.89          3.57          7.15
UNICEF (2021)        |       3.45      3.10          3.83          7.09
UNICEF (2022)        |       2.50      2.27          2.76          7.35
UNICEF (2023)        |       1.72      1.54          1.90          7.46
UNICEF (2024)        |       1.93      1.66          2.24          7.27
 Sub-total           |
  Random pooled  ES  |       2.75      2.17          3.33         50.07
---------------------+---------------------------------------------------
Overall              |
  Random pooled  ES  |       2.75      2.37          3.12        100.00
---------------------+---------------------------------------------------

Test(s) of heterogeneity:
               Heterogeneity  degrees of
                 statistic     freedom           P       I^2**
Boys                   140.26      6           0.00     95.72%
Girls                  162.63      6           0.00     96.31%
Overall                303.07     13           0.00     95.71%
** I^2: the variation in ES attributable to heterogeneity)


Random: Test for heterogeneity between sub-groups:
                         0.00      1           0.98

Significance test(s) of  ES=0

Boys                   z= 9.91       p = 0.00
Girls                  z= 9.28       p = 0.00
Overall                z= 14.35      p = 0.00
-------------------------------------------------------------------------


.
. ge logswt = log(_WT)

. ge logsees = log(_seES)

. **correct plot
. metabias6 _seES  _ES, graph(begg)

Note: default data input format (theta, se_theta) assumed.

Tests for Publication Bias

Begg's Test

  adj. Kendall's Score (P-Q) =      53
          Std. Dev. of Score =   18.27
           Number of Studies =      14
                          z  =    2.90
                 Pr > |z| =   0.004
```

```
                    z  =    2.85 (continuity corrected)
              Pr > |z| =   0.004 (continuity corrected)

Egger's test
------------------------------------------------------------------------
-----
     Std_Eff | Coefficient  Std. err.      t    P>|t|     [95% conf.
interval]
-------------+----------------------------------------------------------
-----
       slope |   .0001427   .0003525     0.40   0.693    -.0006254
.0009107
        bias |   .0555273   .0142085     3.91   0.002     .0245695
.086485
------------------------------------------------------------------------
-----

. metafunnel _seES  _ES

Note: default data input format (theta, se_theta) assumed.

. metabias _seES  _ES, egger

Note: data input format theta se_theta assumed

Egger's test for small-study effects:
Regress standard normal deviate of intervention
  effect estimate against its standard error

.
Number of studies =  14                                Root MSE      =
.0143
------------------------------------------------------------------------
-----
     Std_Eff | Coefficient  Std. err.      t    P>|t|     [95% conf.
interval]
-------------+----------------------------------------------------------
-----
       slope |   .0001427   .0003525     0.40   0.693    -.0006254
.0009107
        bias |   .0555273   .0142085     3.91   0.002     .0245695
.086485
------------------------------------------------------------------------
-----

Test of H0: no small-study effects          P = 0.002

.
.
. graph twoway (lfit logswt year) (scatter logswt year), ///
>        xlabel(2024(1)2018) ///
>   ytitle(log proportion of XXX) ///
>   xtitle(Year of publication)

. pwcorr logswt year, sig

             |   logswt     year
-------------+------------------
      logswt |   1.0000
             |
             |
        year |   0.8210   1.0000
             |   0.0003
```

```
              |

.
.
. ///OTP non-recovery//
>

. metaprop otp_nonrecovery tot_otpexit , by(sex) random  cimethod(exact)
power(2) label
> (namevar=author, yearvar=year)  xlab(0, 1, 2, 3,  4) sortby(year
author) texts(100)
> rflevel(70)

           Study     |      ES       [95% Conf. Interval]  % Weight
---------------------+---------------------------------------------------
     Boys
UNICEF (2018)        |         1.63      1.53         1.74        7.12
UNICEF (2019)        |         3.44      3.32         3.56        7.10
UNICEF (2020)        |         2.02      1.93         2.11        7.14
UNICEF (2021)        |         1.55      1.47         1.63        7.14
UNICEF (2022)        |         1.90      1.83         1.97        7.15
UNICEF (2023)        |         1.16      1.12         1.21        7.17
UNICEF (2024)        |         1.13      1.06         1.20        7.15
 Sub-total           |
  Random pooled  ES  |         1.83      1.37         2.29       49.98
---------------------+---------------------------------------------------
     Girls
UNICEF (2018)        |         1.73      1.64         1.83        7.13
UNICEF (2019)        |         3.30      3.19         3.41        7.12
UNICEF (2020)        |         2.21      2.13         2.30        7.14
UNICEF (2021)        |         1.61      1.54         1.69        7.15
UNICEF (2022)        |         2.06      2.00         2.13        7.16
UNICEF (2023)        |         1.17      1.13         1.21        7.17
UNICEF (2024)        |         1.15      1.09         1.21        7.16
 Sub-total           |
  Random pooled  ES  |         1.89      1.42         2.36       50.02
---------------------+---------------------------------------------------
Overall              |
  Random pooled  ES  |         1.86      1.55         2.17      100.00
---------------------+---------------------------------------------------

Test(s) of heterogeneity:
               Heterogeneity  degrees of
                 statistic     freedom           P       I^2**
Boys                  1570.37      6            0.00     99.62%
Girls                 2024.30      6            0.00     99.70%
Overall               3597.25     13            0.00     99.64%
** I^2: the variation in ES attributable to heterogeneity)


Random: Test for heterogeneity between sub-groups:
                         0.03      1            0.86

Significance test(s) of  ES=0

Boys                  z= 7.83       p = 0.00
Girls                 z= 7.86       p = 0.00
Overall               z= 11.80      p = 0.00
-------------------------------------------------------------------------

.
. ge logswt = log(_WT)
```

```
. ge logsees = log(_seES)

. **correct plot
. metabias6 _seES  _ES, graph(begg)

Note: default data input format (theta, se_theta) assumed.

Tests for Publication Bias

Begg's Test

  adj. Kendall's Score (P-Q) =      33
          Std. Dev. of Score =   18.27
           Number of Studies =      14
                         z  =    1.81
                    Pr > |z| =   0.071
                         z  =    1.75 (continuity corrected)
                    Pr > |z| =   0.080 (continuity corrected)

Egger's test
------------------------------------------------------------------------
-----
     Std_Eff | Coefficient  Std. err.      t    P>|t|     [95% conf.
interval]
-------------+----------------------------------------------------------
-----
       slope |   .0001132   .0000695     1.63   0.129    -.0000382
.0002646
        bias |    .015741   .0044179     3.56   0.004     .0061151
.0253668
------------------------------------------------------------------------
-----

. metafunnel _seES  _ES

Note: default data input format (theta, se_theta) assumed.

. metabias _seES  _ES, egger

Note: data input format theta se_theta assumed

Egger's test for small-study effects:
Regress standard normal deviate of intervention
  effect estimate against its standard error

.
Number of studies =  14                                Root MSE      =
.005
------------------------------------------------------------------------
-----
     Std_Eff | Coefficient  Std. err.      t    P>|t|     [95% conf.
interval]
-------------+----------------------------------------------------------
-----
       slope |   .0001132   .0000695     1.63   0.129    -.0000382
.0002646
        bias |    .015741   .0044179     3.56   0.004     .0061151
.0253668
------------------------------------------------------------------------
-----

Test of H0: no small-study effects          P = 0.004
```

```
.
.
. graph twoway (lfit logswt year) (scatter logswt year), ///
>         xlabel(2024(1)2018) ///
>   ytitle(log proportion of XXX) ///
>   xtitle(Year of publication)

. pwcorr logswt year, sig

             |   logswt     year
-------------+------------------
      logswt |   1.0000 
             |
             |
        year |   0.8531   1.0000 
             |   0.0001
             |

.
. ///SC non-recovery///
>


. metaprop sc_nonnrecovery tot_sc_exit , by(sex) random  cimethod(exact)
power(2) label
> (namevar=author, yearvar=year)  xlab(0, 1, 1.5, 2, 2.5) sortby(year
author) texts(100
> )  rflevel(70)

           Study     |       ES       [95% Conf. Interval]  % Weight
---------------------+---------------------------------------------------
     Boys
UNICEF (2018)        |          1.06      0.77         1.42        6.35
UNICEF (2019)        |          0.98      0.79         1.20        7.13
UNICEF (2020)        |          0.73      0.57         0.91        7.33
UNICEF (2021)        |          0.44      0.32         0.59        7.49
UNICEF (2022)        |          0.59      0.47         0.71        7.55
UNICEF (2023)        |          0.46      0.37         0.57        7.63
UNICEF (2024)        |          2.22      1.91         2.56        6.30
 Sub-total           |
  Random pooled  ES  |          0.90      0.61         1.18       49.78
---------------------+---------------------------------------------------
     Girls
UNICEF (2018)        |          1.38      1.06         1.77        6.10
UNICEF (2019)        |          0.76      0.59         0.96        7.26
UNICEF (2020)        |          0.77      0.61         0.95        7.32
UNICEF (2021)        |          0.25      0.16         0.36        7.62
UNICEF (2022)        |          0.53      0.42         0.65        7.57
UNICEF (2023)        |          0.45      0.36         0.55        7.64
UNICEF (2024)        |          1.65      1.40         1.93        6.72
 Sub-total           |
  Random pooled  ES  |          0.80      0.53         1.06       50.22
---------------------+---------------------------------------------------
Overall              |
  Random pooled  ES  |          0.84      0.66         1.02      100.00
---------------------+---------------------------------------------------

Test(s) of heterogeneity:
               Heterogeneity  degrees of
                 statistic     freedom          P       I^2**
Boys                 137.12       6            0.00     95.62%
Girls                143.31       6            0.00     95.81%
Overall              287.02      13            0.00     95.47%
```

```
** I^2: the variation in ES attributable to heterogeneity)


Random: Test for heterogeneity between sub-groups:
                         0.25      1           0.61

Significance test(s) of  ES=0

Boys                   z= 6.21       p = 0.00
Girls                  z= 5.93       p = 0.00
Overall                z= 9.03       p = 0.00
-------------------------------------------------------------------------

.
. ge logswt = log(_WT)

. ge logsees = log(_seES)

. **correct plot
. metabias6 _seES  _ES, graph(begg)

Note: default data input format (theta, se_theta) assumed.

Tests for Publication Bias

Begg's Test

  adj. Kendall's Score (P-Q) =      69
          Std. Dev. of Score =   18.27
           Number of Studies =      14
                          z  =    3.78
                 Pr > |z| =   0.000
                          z  =    3.72 (continuity corrected)
                 Pr > |z| =   0.000 (continuity corrected)

Egger's test
-------------------------------------------------------------------------
-----
     Std_Eff | Coefficient  Std. err.      t    P>|t|     [95% conf.
interval]
-------------+-----------------------------------------------------------
-----
       slope |   .0002478   .0000705     3.52   0.004     .0000942
.0004014
        bias |   .0791205   .0128149     6.17   0.000     .0511991
.1070418
-------------------------------------------------------------------------
-----

. metafunnel _seES  _ES

Note: default data input format (theta, se_theta) assumed.

. metabias _seES  _ES, egger

Note: data input format theta se_theta assumed

Egger's test for small-study effects:
Regress standard normal deviate of intervention
  effect estimate against its standard error

.
Number of studies =  14                              Root MSE      =
```

```
.0239
------------------------------------------------------------------------------
-----
     Std_Eff | Coefficient  Std. err.      t    P>|t|     [95% conf.
interval]
-------------+----------------------------------------------------------------
-----
       slope |   .0002478   .0000705     3.52   0.004     .0000942
.0004014
        bias |   .0791205   .0128149     6.17   0.000     .0511991
.1070418
------------------------------------------------------------------------------
-----

Test of H0: no small-study effects          P = 0.000

.
.
. graph twoway (lfit logswt year) (scatter logswt year), ///
>         xlabel(2024(1)2018) ///
>   ytitle(log proportion of XXX) ///
>   xtitle(Year of publication)

. pwcorr logswt year, sig

             |   logswt     year
-------------+------------------
      logswt |   1.0000
             |
             |
        year |   0.2657   1.0000
             |   0.3585
             |

.
. ///SC transfers///

. metaprop sc_transfers tot_sc_exit , by(sex) random  cimethod(exact)
power(2) label(na
> mevar=author, yearvar=year)  xlab(0, 5, 10, 15, 20, 25) sortby(year
author) texts(100
> )  rflevel(70)

           Study     |      ES       [95% Conf. Interval]  % Weight
---------------------+--------------------------------------------------
     Boys
UNICEF (2018)        |         24.95     23.64        26.30          6.80
UNICEF (2019)        |         13.82     13.13        14.54          7.16
UNICEF (2020)        |         10.97     10.37        11.59          7.19
UNICEF (2021)        |         13.90     13.22        14.59          7.17
UNICEF (2022)        |         12.17     11.67        12.68          7.23
UNICEF (2023)        |         12.40     11.94        12.87          7.24
UNICEF (2024)        |         10.39      9.74        11.07          7.17
 Sub-total           |
  Random pooled  ES  |         14.01     11.98        16.05         49.96
---------------------+--------------------------------------------------
     Girls
UNICEF (2018)        |         22.49     21.27        23.76          6.86
UNICEF (2019)        |         12.83     12.15        13.53          7.16
UNICEF (2020)        |         10.60     10.02        11.20          7.20
UNICEF (2021)        |         14.23     13.54        14.94          7.16
UNICEF (2022)        |         12.99     12.48        13.53          7.22
UNICEF (2023)        |         12.33     11.88        12.79          7.24
```

```
UNICEF (2024)        |        10.26      9.64        10.90        7.19
 Sub-total           |
  Random pooled  ES  |        13.62     11.77        15.46       50.04
---------------------+----------------------------------------------------
Overall              |
  Random pooled  ES  |        13.81     12.50        15.11      100.00
---------------------+----------------------------------------------------

Test(s) of heterogeneity:
               Heterogeneity  degrees of
                 statistic     freedom          P       I^2**
Boys                   438.09      6            0.00     98.63%
Girls                  371.62      6            0.00     98.39%
Overall                809.97     13            0.00     98.39%
** I^2: the variation in ES attributable to heterogeneity)


Random: Test for heterogeneity between sub-groups:
                         0.08      1            0.78

Significance test(s) of  ES=0

Boys                  z= 13.53      p = 0.00
Girls                 z= 14.45      p = 0.00
Overall               z= 20.73      p = 0.00
--------------------------------------------------------------------------

.
. ge logswt = log(_WT)

. ge logsees = log(_seES)

. **correct plot
. metabias6 _seES  _ES, graph(begg)

Note: default data input format (theta, se_theta) assumed.

Tests for Publication Bias

Begg's Test

  adj. Kendall's Score (P-Q) =      39
          Std. Dev. of Score =   18.27
           Number of Studies =      14
                          z  =    2.14
                 Pr > |z| =   0.033
                          z  =    2.08 (continuity corrected)
                 Pr > |z| =   0.037 (continuity corrected)

Egger's test
--------------------------------------------------------------------------
-----
     Std_Eff | Coefficient  Std. err.      t    P>|t|     [95% conf.
interval]
-------------+------------------------------------------------------------
-----
       slope |    .000334   .0007077     0.47   0.645     -.001208
.001876
        bias |   .0229001   .0055708     4.11   0.001     .0107624
.0350378
--------------------------------------------------------------------------
-----
```

```
. metafunnel _seES  _ES

Note: default data input format (theta, se_theta) assumed.

. metabias _seES  _ES, egger

Note: data input format theta se_theta assumed

Egger's test for small-study effects:
Regress standard normal deviate of intervention
  effect estimate against its standard error

.
Number of studies =  14                              Root MSE      =
.0044
--------------------------------------------------------------------------
-----
     Std_Eff | Coefficient  Std. err.      t    P>|t|     [95% conf.
interval]
-------------+------------------------------------------------------------
-----
       slope |    .000334   .0007077     0.47   0.645     -.001208
.001876
        bias |   .0229001   .0055708     4.11   0.001     .0107624
.0350378
--------------------------------------------------------------------------
-----

Test of H0: no small-study effects          P = 0.001

.
.
. graph twoway (lfit logswt year) (scatter logswt year), ///
>         xlabel(2024(1)2018) ///
>   ytitle(log proportion of XXX) ///
>   xtitle(Year of publication)

. pwcorr logswt year, sig

             |   logswt     year
-------------+------------------
      logswt |   1.0000 
             |
             |
        year |   0.6750   1.0000 
             |   0.0081
             |

.
end of do-file

--------------------------------------------------------------------------
--------------
```
